# Genetic Evidence for Elevated Pathogenicity of Mitochondrial DNA Heteroplasmy in Autism Spectrum Disorder

**DOI:** 10.1371/journal.pgen.1006391

**Published:** 2016-10-28

**Authors:** Yiqin Wang, Martin Picard, Zhenglong Gu

**Affiliations:** 1 Division of Nutritional Sciences, Cornell University, Ithaca, New York, United States of America; 2 Department of Psychiatry, Division of Behavioral Medicine, Columbia University Medical Center, New York, New York, United States of America; 3 Department of Neurology, Division of Columbia Translational Neuroscience Initiative, Columbia University Medical Center, New York, New York, United States of America; Pennsylvania State University, UNITED STATES

## Abstract

Increasing clinical and biochemical evidence implicate mitochondrial dysfunction in the pathophysiology of Autism Spectrum Disorder (ASD), but little is known about the biological basis for this connection. A possible cause of ASD is the genetic variation in the mitochondrial DNA (mtDNA) sequence, which has yet to be thoroughly investigated in large genomic studies of ASD. Here we evaluated mtDNA variation, including the mixture of different mtDNA molecules in the same individual (i.e., heteroplasmy), using whole-exome sequencing data from mother-proband-sibling trios from simplex families (n = 903) where only one child is affected by ASD. We found that heteroplasmic mutations in autistic probands were enriched at non-polymorphic mtDNA sites (*P* = 0.0015), which were more likely to confer deleterious effects than heteroplasmies at polymorphic mtDNA sites. Accordingly, we observed a ~1.5-fold enrichment of nonsynonymous mutations (*P* = 0.0028) as well as a ~2.2-fold enrichment of predicted pathogenic mutations (*P* = 0.0016) in autistic probands compared to their non-autistic siblings. Both nonsynonymous and predicted pathogenic mutations private to probands conferred increased risk of ASD (Odds Ratio, OR[95% CI] = 1.87[1.14–3.11] and 2.55[1.26–5.51], respectively), and their influence on ASD was most pronounced in families with probands showing diminished IQ and/or impaired social behavior compared to their non-autistic siblings. We also showed that the genetic transmission pattern of mtDNA heteroplasmies with high pathogenic potential differed between mother-autistic proband pairs and mother-sibling pairs, implicating developmental and possibly *in utero* contributions. Taken together, our genetic findings substantiate pathogenic mtDNA mutations as a potential cause for ASD and synergize with recent work calling attention to their unique metabolic phenotypes for diagnosis and treatment of children with ASD.

## Author Summary

Mitochondria contain their own genome, the mitochondrial DNA (mtDNA), and are abundant in the brain where they produce energy and intracellular signals required for normal brain function and cognition. Mitochondrial dysfunction has been proposed as a cause of Autism Spectrum Disorder (ASD), but the genetic basis has not been established. By analyzing mtDNA sequences from 903 ASD children along with their unaffected siblings and mothers, we found a unique pattern of heteroplasmic mtDNA mutations (the coexistence of both mutant and wide-type mtDNA molecules in a cell) associated with increased risk of ASD. A large fraction of these mtDNA heteroplasmies are pathogenic and can be either inherited maternally or acquired during development. Our findings unveil an important issue about pathogenic mtDNA heteroplasmy transmission between mothers and children, which could underlie many neurodevelopmental disorders in children that are shown to involve mitochondrial dysfunction. Evaluating mtDNA heteroplasmies in high-risk families could help diagnosis and treatment of these diseases.

## Introduction

Autism Spectrum Disorder (ASD) refers to a broad collection of complex, neurodevelopmental disorders, characterized by impairment of communicative and social interactions [1]. ASD usually manifests at an early stage of development in pre-pubertal children [2]. The current prevalence of ASD in the United States according to the 2014 annual report from The Centers for Disease Control and Prevention (CDC) is 1 in 68 children (1.47%), with a skewed gender ratio of 4 affected boys to 1 affected girl [3]. Population-based studies suggest an even higher prevalence of 2.24% in the United States [4]. Treatment for ASD mostly relies on behavioral interventions with specialized training to lessen social, verbal and cognitive deficits. However, the effectiveness varies widely among children with ASD and mild forms of symptoms such as attention deficit and social difficulties may persist throughout life [5].

Although ASD is traditionally described as a developmental disorder of the central nervous system, emerging evidence suggests that systemic physiological abnormalities, including dysregulated inflammation and immune system [6,7], elevated oxidative stress [8], and mitochondrial dysfunction [9–12], are present in peripheral tissues as well as in brains of autistic patients [13]. Epidemiological studies also identified a staggeringly high comorbidity between ASD and mitochondrial disorder (MD) [14]. MD is a heterogeneous group of diseases due to maternally inherited or sporadic defects in genes encoding the mitochondrial oxidative phosphorylation (OXPHOS) system [15], the core functional component of mitochondria responsible for generating energy. MD usually results from genetic mutations on nuclear DNA or mitochondrial DNA (mtDNA) genes [15]. It has an extremely low birth prevalence of 6.2 in 100,000 children [16] and only about 1 in 4,300 adults is affected or at risk of developing MD [17]. However, the incidence of MD among autistic patients is estimated up to 5% by some studies [14], over 200-fold the incidence of MD in general populations. In addition, expression of OXPHOS genes has been shown to decrease in brains of autistic patients [18], suggesting a biological overlap in the pathogenesis of MD and ASD. Moreover, decreased activity of the five OXPHOS complexes has been observed in leukocytes, buccal cells, muscle biopsies and brains of autistic patients [11,14,19], suggesting the increased MD incidence among autistic patients is due not to a specific protein defect but to defects across components of the OXPHOS system.

Notwithstanding ever-growing evidence implicating mitochondrial dysfunction in the pathophysiology of ASD, little is known about the biological basis of this connection [19]. Polymorphisms in genes in mitochondria-related pathways, such as *PARK2* [20] and *SLC25A12* [21], were previously found to be associated with ASD in genome-wide or candidate gene association studies, suggesting a genetic basis. However, in recent comprehensive genetic studies among over two thousand affected families, few of the identified risk loci for ASD harbor genes showing a direct link with mitochondrial function [22–25]. The results from mtDNA studies are also mixed. A number of studies demonstrated elevation of mtDNA deletions [10,26], point mutations [9,26,27], or copy numbers [10,28] in autistic patients. Interestingly, increased mtDNA copy number can occur secondarily to mitochondrial defects [29], further indicating the presence of genetic and/or functional defects in ASD. But few of these findings have been confirmed in large populations [30].

Because there are thousands of mtDNA molecules in a single cell, new mutations can arise and coexist with the wide-type mtDNA in a state called heteroplasmy, which has been implicated in a wide range of diseases [31,32]. To evaluate mtDNA heteroplasmy among healthy individuals, next-generation sequencing technologies such as mtDNA-targeted sequencing [33–36] or computational approaches, which directly leverage (off-target) reads from existing datasets produced by whole-genome sequencing [37,38] or whole-exome sequencing [39,40] have been widely harnessed in recent years. These approaches largely outperform Sanger sequencing and microarray-based sequencing in their ability to detect mtDNA heteroplasmies, and have proven practical for large population-based studies [37–39].

In the current study, we investigated the connection between mtDNA variation, especially heteroplasmy, and ASD by leveraging off-target reads from exome-sequencing data sets among simplex families from the Simons Foundation Autism Research Initiative (SFARI) Simons Collection deposited at the National Autism Research Database [23]. Each simplex family is composed of one autistic proband, his or her non-autistic sibling and their parents. Because the proband and the sibling have the same mtDNA background, share family environment, and are of comparable age, our study avoids genetic and environmental confounding factors that have limited the interpretation of studies among unrelated case-control pairs or child-parent pairs [30]. Given that the mtDNA is maternally inherited, we analyzed data for mother-proband-sibling trios from 903 simplex families and utilized stringent criteria to determine mtDNA homoplasmies and heteroplasmies. We then compared mtDNA variation between autistic probands and non-autistic siblings, along with the transmission pattern of mtDNA mutations between the mother and the child. We showed that autistic children have overrepresented pathogenic mutations on mtDNA distinct from their unaffected siblings. Our results offer an important angle to explain mitochondrial dysfunction in ASD patients, pointing towards the accumulation of mtDNA mutations of high pathogenic potential during development as one possible mechanism underlying the metabolic pathophysiology of ASD.

## Results

### Characteristics of mtDNA variants in the studied families

The whole-exome sequencing data for the SFARI Simons Collection were generated by three genome centers including CSHL (Cold Spring Harbor Laboratory), YALE (the Department of Genetics at the Yale School of Medicine) and UW (Genome Sciences at the University of Washington) [23]. Only reads generated by CSHL met our minimal mtDNA sequence coverage depth, where reads from YALE and UW yielded on average less than 20% of mtDNA sites sequenced to >20X depth (S1 Fig). Hence, this study focused on the 933 families sequenced at CSHL. After quality control, we obtained data for mother-proband-sibling trios from 903 families, or 2,709 individuals. The average depth of sequencing coverage on mtDNA was 141X, which allowed us to call homoplasmies and medium-fraction heteroplasmies.

We first examined the distribution of mtDNA variants. In total, we identified 68,670 homoplasmies according to the revised Cambridge Reference Sequence (rCRS) at 2,152 mtDNA polymorphic sites. Among them, up to 1,847 (85.8%) sites showed the minor allele in less than 1% of individuals, and 1,031 (47.9%) sites were unique to individual families (S2 Fig). When filtering high-confidence heteroplasmies at minor allele fraction (MAF) ≥5%, we identified 677 heteroplasmies at 366 mtDNA sites. Based on this stringent criterion to define heteroplasmy, approximately 21.1% of individuals carried at least one heteroplasmy on mtDNA. Individual (n = 229, 62.6%) or family (n = 323, 88.3%) specific heteroplasmies contributed to the majority of the heteroplasmic sites detected (S2 Fig). Only one heteroplasmy, located at position 16,093 in the D-loop region, was present in ≥1% of individuals.

**Fig 1:**
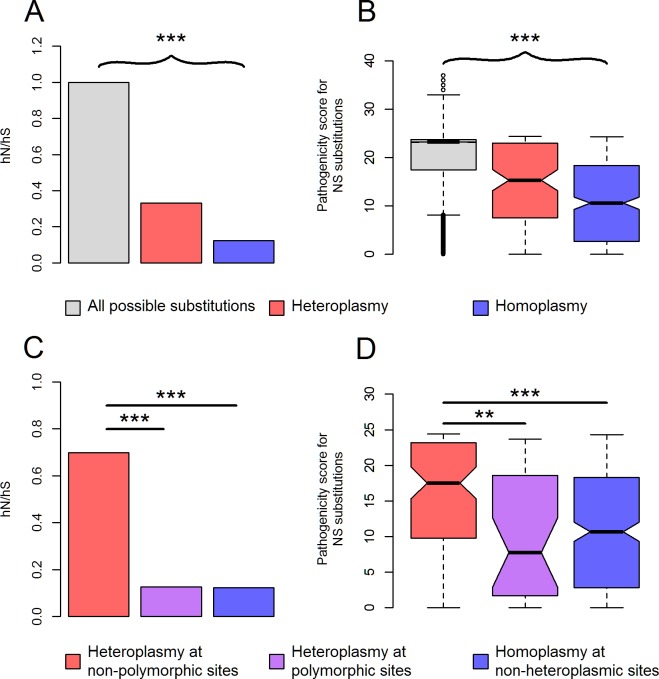
Characteristics of mtDNA variants. **(A)** The nonsynonymous-synonymous rate ratio (hN/hS) for all possible nucleotide substitutions, observed heteroplasmies, and homoplasmies on mtDNA. hN is the number of nonsynonymous substitutions divided by the total number of possible nonsynonymous substitutions on mtDNA; similarly, hS is the number of synonymous substitutions divided by the total number of possible synonymous substitutions on mtDNA [34]. The ratio of hN and hS is indicative of purifying selection if it is significantly less than one [34]. **(B)** The box plot of CADD pathogenicity scores for all possible substitutions, observed heteroplasmies and homoplasmies at nonsynonymous sites on mtDNA. **(C, D)** hN/hS **(C)** and CADD pathogenicity scores of nonsynonymous substitutions **(D)** for heteroplasmies at non-polymorphic sites, heteroplasmies at polymorphic sites and homoplasmies at non-heteroplasmic sites. Each nucleotide substitution was only counted at most once. ***P<2x10^-4^; **P<2x10^-3^; *P*: p values for Chi-squared test **(A,C)** or Mann-Whitney test **(B,D)**.

Consistent with our previous findings in the 1000 Genomes population [37,41], we found strong evidence to support the extensive purifying selection on mtDNA. For both homoplasmies and heteroplasmies, their nonsynonymous-synonymous rate ratio was significantly less than one (*P*<5x10^-19^, Fig 1A); their pathogenic potential at nonsynonymous sites was considerably lower than that of all possible, nonsynonymous nucleotide substitutions on mtDNA (*P*<2x10^-14^, Fig 1B). Of note, compared to homoplasmies, heteroplasmies exhibited higher nonsynonymous-synonymous rate ratio (*P* = 1.2x10^-11^, Fig 1A), along with elevated pathogenicity at nonsynonymous sites (*P* = 1.5x10^-4^, Fig 1B). This may be due to mitochondrial threshold effect [42], which masks deleterious phenotypes of low-fraction mtDNA heteroplasmies, allowing them to persist without being purged by natural selection.

We found that 194 (53.0%) heteroplasmic sites were located at polymorphic sites. Interestingly, only heteroplasmies at non-polymorphic sites exhibited inflated pathogenicity compared to homoplasmies (*P*<1.7x10^-6^, Fig 1C and 1D). mtDNA heteroplasmies at non-polymorphic sites also possessed very low relative mutation rates (S3 Fig). Both features impeded the introduction of these mutations into the phylogenetic tree; low mutation rates were responsible for the rare occurrence of these mutations in the first place; if they did mutate and appeared initially in the heteroplasmic state, purifying selection would act on them as soon as the mutant allele fraction exceeded the mitochondrial threshold for manifesting the associated, deleterious phenotype affecting survival or reproduction. The opposite case lies in the mtDNA mutations that have been shown to have entered the phylogenetic tree multiple times, demonstrating low pathogenicity and high relative mutation rates [43]. In comparison with homoplasmies at non-heteroplasmic sites, heteroplasmies at polymorphic sites exhibited similar level of pathogenicity (Fig 1C and 1D), but were more likely to mutate (Mann-Whitney test, *P* = 8x10^-32^, S3 Fig), suggesting their high incidence of being heteroplasmic may be primarily the consequence of their inherent, high mutation rates.

### Elevated pathogenicity of mtDNA mutations in autistic probands

**Fig 2:**
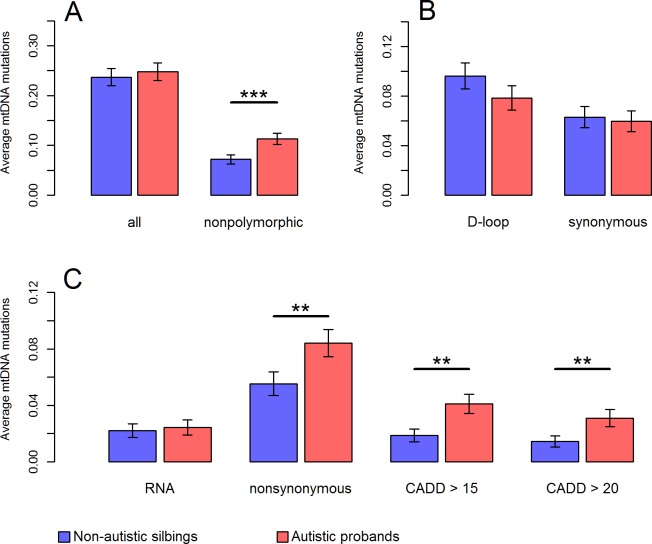
Comparison of mtDNA mutation burden and pathogenicity between autistic probands and non-autistic siblings. Mutation burden was calculated using mutations with DAF ≥5% at mtDNA sites where all members in a family had >40X sequencing depth. The average number of mutations per individual was shown in **(A)** for all valid sites and non-polymorphic sites, in **(B)** for sites in the D-loop region and synonymous sites in the coding region, and in **(C)** for sites in the RNA region, nonsynonymous sites in the coding region, and mutations predicted pathogenic with CADD Phred score >15 or >20. Results in the main text were based on “CADD > 15”. Error bars represent the standard error of the mean. ***P<0.001; **P<0.01; *P<0.05; *P*: p values for one-tailed paired t-test.

Given evidence on mitochondrial implication in ASD, we postulated that autistic probands might carry mutation burden on mtDNA, especially mutations prone to confer deleterious effects on OXPHOS. To test this hypothesis, we directly compared the number as well as the distribution of mtDNA mutations, at segregating sites within each family, between autistic probands and their non-autistic siblings. For this comparison, we analyzed mtDNA sites where all members in a family had >40X sequencing depth. Among the 903 families, 191 autistic probands (21.2%) and 182 non-autistic siblings (20.2%) carried at least one high-confidence heteroplasmic or homoplasmic mutation with derived allele fraction (DAF) ≥5% (referred to throughout as mutation). On average, autistic probands carried 0.25 mutations and their non-autistic siblings carried 0.24 mutations, indicating the overall mutation burden was not significantly different between these two groups (*P* = 0.3, Fig 2A). However, heteroplasmic mutations were more likely to be located at non-polymorphic sites in autistic probands than those in non-autistic siblings (Chi-squared test, *P* = 0.0015). The incidence of heteroplasmic mutations at non-polymorphic sites was 53% higher in autistic probands compared to non-autistic siblings (0.11 per proband vs. 0.072 per sibling, *P* = 0.0003, Fig 2A).

Because the observed pathogenicity of mtDNA mutations was primarily attributed to heteroplasmies at non-polymorphic sites (Fig 1C and 1D), overrepresented mutations at non-polymorphic sites might imply an elevated pathogenicity. As expected, further stratifying mutations according to their functional annotations revealed that autistic probands carried 52% more nonsynonymous mutations (0.084 per proband vs. 0.055 per sibling; *P* = 0.0028, Fig 2C), and 118% more predicted pathogenic mutations compared to non-autistic siblings (0.041 per proband vs. 0.019 per sibling; *P* = 0.0016, Fig 2C). Autistic probands also had roughly twice as many mutations associated with disease as their non-autistic siblings had (0.012 per proband vs. 0.006 per sibling; *P* = 0.017, S4 Fig). Additionally, the observed increases of pathogenic mutations in autistic probands were insensitive to changes in minimum DAF we used for defining mutations (from 2% to 8%, S5 Fig). In line with these results, there was no significant evidence on mutation burden at synonymous sites as well as in the D-loop region (*P*>0.6, Fig 2B), which were normally considered to harbor relatively neutral variants [44].

There were 265 mtDNA mutations that were private to only one child within a family: its derived allele was undetectable or had fraction <2% in the other child. Carrying nonsynonymous private mutations was associated with an increased risk of ASD (Odds Ratio, OR: 1.87, *P* = 0.0055, Table 1). Likewise, carrying private mutations of predicted pathogenicity more than doubled the risk of ASD (OR: 2.55, *P* = 0.0036, Table 1). Among the 596 families whose ASD risk was not attributed to any *de novo* likely gene-disruptive (LGD) SNVs or CNVs in the nuclear genomes [23,24], we attained comparable ORs for ASD risk at 2.00 and 2.42 for nonsynonymous private mutations and pathogenic private mutations, respectively (*P*<0.025, Table 1). Therefore, the observed association between mtDNA mutations and ASD is unlikely to be contingent upon spontaneous nuclear gene mutations.

**Table 1:** Contribution of private mtDNA mutations to ASD.

Subpopulations	Groups	Proband ^a^	Sibling ^a^	OR [95% CI]	*P*
All	Nonsynonymous	51 (5.6%)	28 (3.1%)	1.87 [1.14–3.11]	0.0055
(n = 903 families)	CADD > 15	30 (3.3%)	12 (1.3%)	2.55 [1.26–5.51]	0.0036
	CADD > 20	22 (2.4%)	9 (1.0%)	2.48 [1.09–6.15]	0.014
No *de novo* LGD variants ^b^	Nonsynonymous	35 (5.9%)	18 (3.0%)	2.00 [1.09–3.80]	0.012
(n = 596 families)	CADD > 15	19 (3.2%)	8 (1.3%)	2.42 [1.00–6.44]	0.025
	CADD > 20	16 (2.7%)	7 (1.2%)	2.32 [0.89–6.72]	0.044
Normal IQ	Nonsynonymous	20 (4.7%)	16 (3.7%)	1.26 [0.61–2.64]	0.31
(n = 428 families) ^c^	CADD > 15	12 (2.8%)	6 (1.4%)	2.03 [0.70–6.65]	0.12
	CADD > 20	7 (1.6%)	5 (1.2%)	1.41 [0.38–5.66]	0.39
Diminished IQ	Nonsynonymous	28 (6.6%)	10 (2.3%)	2.92 [1.36–6.84]	0.0021
(n = 427 families) ^c^	CADD > 15	16 (3.7%)	5 (1.2%)	3.28 [1.14–11.6]	0.012
	CADD > 20	13 (3.0%)	4 (0.9%)	3.32 [1.01–14.1]	0.023
Concordant SRS	Nonsynonymous	22 (5.5%)	17 (4.2%)	1.31 [0.65–2.67]	0.26
(n = 403 families) ^d^	CADD > 15	15 (3.7%)	8 (2.0%)	1.91 [0.75–5.26]	0.10
	CADD > 20	11 (2.7%)	5 (1.2%)	2.23 [0.71–8.27]	0.10
Discordant SRS	Nonsynonymous	29 (5.9%)	10 (2.0%)	3.02 [1.41–7.02]	0.0014
(n = 492 families) ^d^	CADD > 15	15 (3.0%)	4 (0.8%)	3.83 [1.21–16.0]	0.0090
	CADD > 20	11 (2.2%)	4 (0.8%)	2.79 [0.82–12.1]	0.058

^a^Data are the number of individuals carrying private mutations (percentage of families).

^b^families whose ASD risk was not attributed to *de novo* LGD SNVs or CNVs; data on exomic *de novo* LGD SNPs and CNVs were obtained from references [23,24].

^c^families with probands having full-scale IQ >85 or ≤85; IQ information for 48 families was not available.

^d^Families with discordant SRS scores had proband SRS score >75 and sibling SRS score <50; otherwise they were considered as having concordant SRS scores; proband and sibling SRS scores were not available in 8 families.

CADD: mutations predicted pathogenic with CADD Phred score >15 or >20; results in the main text were based on “CADD > 15”; *P*: one-tailed p values for Fisher's exact test.

### Pathogenic mtDNA mutations correlate with ASD-associated phenotypes

We further sought to investigate the relationship between mtDNA mutations and ASD-associated phenotypes, including cognitive ability and social behavior. Compared to ORs in total families, the ORs for ASD risk were attenuated when estimated only in families with the proband classified as having normal intelligence quotient (full-scale IQ >85, referred to throughout as IQ) (Table 1). A similar attenuated genetic contribution to ASD was also identified in previous nuclear genome studies [23,24], suggesting a predominant role of genetic factors in the pathogenesis of ASD only in the context of diminished IQ. Next, we retrieved the Social Responsiveness Scale scores for probands and siblings (parent-reported total SRS T scores, referred to throughout as SRS) in the 903 families, which is a measure of ASD severity in natural social settings. We defined families with discordant proband-sibling social behavior using criteria according to Krumm *et al*. [24] where autistic probands demonstrated severe social impairments (SRS score >75) as opposed to their non-autistic siblings (SRS score <50). We found that ORs of mtDNA mutations for ASD risk were stronger in families with discordant SRS scores between probands and siblings than families with concordant SRS scores (Table 1), indicating that mtDNA mutations might segregate with autistic behavioral phenotypes at a family level.

Because behavioral impairment inversely correlated with cognitive ability in autistic probands (Pearson correlation coefficient between SRS scores and IQ, *r* = -0.22, *P* = 4.5x10^-11^), we further stratified the 903 families into four groups according to the criteria on both IQ and SRS scores. Even though the sample sizes were getting smaller in each category, the effects of mtDNA private mutations on ASD were most significant (ORs = 3.56–4.61, Fisher’s exact test, *P*<0.09, S1 Table) in families with discordant proband-sibling SRS scores and diminished proband IQ, and were least pronounced (ORs = 0.90–1.77, Fisher’s exact test, *P*>0.27, S1 Table) in families with concordant SRS scores and normal proband IQ, suggesting that the observed contribution of mtDNA mutations to ASD was mostly attributed to families with probands showing noticeable deficits in cognition and/or social interactions distinct from their siblings.

Of note, the incidence of private mtDNA mutations was consistently higher in autistic probands of the diminished IQ group than that of the normal IQ group (3.0%–6.6% vs. 1.6%–4.7%, Table 1). To examine whether carrying private mtDNA mutations affects cognitive ability, we further analyzed the impact of private mtDNA mutations on IQ as well as intellectual disability (ID, which we defined as IQ <70) among autistic probands. In the 903 families, proband IQ was significantly associated with family ancestry (one-way ANOVA, *P*<4.8x10^-10^). To avoid such confounding factors on proband IQ comparisons among families, we focused on 638 probands of the major ethnic group in the current study for further analysis (S6 Fig and Table 2). Private mtDNA mutations detected at non-polymorphic sites were significantly associated with decreased proband IQ (linear regression, **β** = -7.8, *P* = 0.02, S6 Fig). Similar effects on IQ were also observable for nonsynonymous and predicted pathogenic mtDNA mutations private to probands, but the corresponding *P* values were not significant (**β** = -7.3 –-5.6, *P* = 0.15–0.27, S6 Fig). A considerable decrease in IQ was identified in probands with disease-associated private mtDNA mutations which lowered the average IQ by approximately 25 points compared to those devoid of these mutations (*P* = 0.01, S6 Fig).

**Table 2:** Contribution of private mtDNA mutations to ID in ASD.

Groups	Model 1^a^		Model 2 ^a^	
	OR [95% CI]	*P*	OR [95% CI]	*P*
Non-polymorphic	2.34 [1.36–4.03]	0.0022	2.41 [1.39–4.18]	0.0018
Polymorphic	1.11 [0.62–1.98]	0.72	1.06 [0.59–1.89]	0.85
Nonsynonymous	1.98 [1.03–3.78]	0.039	1.89 [0.98–3.66]	0.058
CADD > 15	2.42 [1.09–5.37]	0.031	2.35 [1.05–5.28]	0.039
CADD > 20	2.75 [1.10–6.85]	0.030	2.59 [1.04–6.45]	0.040
Disease-associated	2.59 [0.57–11.9]	0.22	2.38 [0.51–11.1]	0.27

Associations of ID (defined as full-scale IQ <70) with proband-private mtDNA mutations were computed in 638 probands of the major ethnic group among the 903 families who had a reported family ancestry of “white” or “more-than-one-race” and an mtDNA-haplogroup inferred maternal ancestry of “Europe”.

^a^OR (odds ratio), 95% CI (confidence interval) of OR, and *P* values were computed for mutations in each category using logistic regression Model 1 (adjusting for age and sex) and Model 2 (adjusting for age, sex, SRS scores and whether carrying *de novo* LGD SNPs or CNVs on nuclear DNA).

CADD: mutations predicted pathogenic with CADD Phred score >15 or >20; results in the main text were based on “CADD > 15”.

We then asked whether the observed shift of IQ distribution could lead to comorbidity of ASD and ID by using logistic regression. We found that mtDNA mutations at non-polymorphic sites as well as nonsynonymous and predicted pathogenic mtDNA mutations that were private to probands significantly increased the risk of ID in ASD (ORs = 1.98–2.75, *P* = 0.0022–0.039, Table 2), The estimated ORs did not qualitatively change after additional adjustment for ASD severity (proband SRS scores) and whether carrying *de novo* LGD SNPs or CNVs on nuclear DNA (Table 2). It suggests that mtDNA mutations and the resulting mitochondrial dysfunction may be responsible for intellectual disability in ASD, which is consistent with clinical reports on ASD-ID cases who manifested defects in mitochondrial OXPHOS [45].

### Different mother-to-child mtDNA mutation transmission between autistic probands and non-autistic siblings

Because mtDNA is uniquely inherited from the mother [46], this dataset also provided us a great opportunity to investigate the transmission pattern of mtDNA mutations from mothers to children. Among all inherited events of homoplasmies from the mother to the child, 99.3% (45,488) were direct transmissions of maternal homoplasmies, and the remaining 0.7% of events resulted from the fixation of either the major allele (n = 295) or the minor allele (n = 6) of maternal heteroplasmies. Among all mother-child pairs, we only identified one possible *de novo* homoplasmy in a non-autistic sibling (family id: 12899), located in the D-loop region (143G>A), whose mutant allele A was not detected in either the mother or the autistic proband.

There was a significant correlation of derived allele fractions for heteroplasmies between the mother and the child (Pearson correlation coefficient *r* = 0.38, *P* = 1.9x10^-10^). For heteroplasmies with MAF ≥ 5% in the child, 53.1% were shared with the mother, and therefore were inherited, in agreement with previous studies [36,38]. The proportion of inherited heteroplasmies decreased to 33.7%, when we lowered the minimum MAF in calling heteroplasmies in children to 2% (S7 Fig), indicating that a majority of heteroplasmies in children could be of developmental origin. However, we might underestimate the proportion of inherited heteroplasmies, since some heteroplasmies might be of low fraction or somatically lost in the mother.

**Fig 3:**
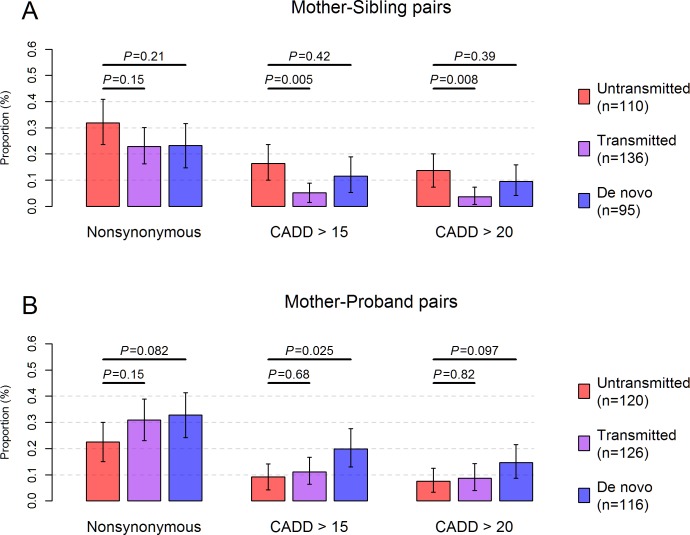
Transmission pattern of pathogenic mtDNA mutations. The proportion of untransmitted (red), transmitted (purple) and *de novo* (blue) mutations that were nonsynonymous or predicted pathogenic was shown in (**A**) for mother-sibling pairs and in (**B)** for mother-proband pairs. Only mtDNA sites (n = 448) detected with high-confidence heteroplasmies (MAF ≥5%) or *de novo* homoplasmies were used for calculation. Untransmitted mutations were defined as mutations present in the mother, but were undetectable or had DAF <2% in the child. Likewise, *de novo* mutations were defined as those present in the child, but were undetectable or had DAF <2% in the mother. Transmitted mutations were mutations shared between the mother and the child with DAF ≥2%. The total number of untransmitted, transmitted and *de novo* mutations was indicated in parentheses for mother-sibling pairs and mother-proband pairs in the legend. Error bars represent the 95% confidence interval of the proportion estimated based on 10,000 bootstrap samples. CADD: mutations predicted pathogenic with CADD Phred score >15 or >20. Results in the main text were based on “CADD > 15”; *P*: p values for Fisher’s exact test.

It has been shown that purifying selection operates during oogenesis and early embryogenesis to reduce the possibility of pathogenic mtDNA mutations being transmitted to the subsequent generation [47]. This mtDNA quality control mechanism ensures the OXPHOS function in the maternal germ line and thereby in the child [48]. The influence of this purification selection process was evidenced in our dataset with a significant decrease in the proportion of transmitted mutations that were predicted pathogenic, compared to that of untransmitted mutations in mother-sibling pairs (*P* = 0.005, Fig 3A). However, evidence of the purification selection disappeared when computed in mother-autistic proband pairs, which might be skewed by the ascertainment of disease status in autistic probands [49] (Fig 3B). Interestingly, *de novo* mutations in autistic probands showed an even higher proportion of predicted pathogenic mutations compared to untransmitted mutations (*P* = 0.025, Fig 3B) in mother-autistic proband pairs.

To ascertain the pathogenic mutation burden observed in autistic probands, we further calculated the changes of derived allele fractions between mutations in the mother and mutations in the child (ΔDAF = DAF_child_–DAF_mother_). Overall, ΔDAF followed a normal distribution centered about zero for mutations in both mother-sibling pairs and mother-proband pairs (S8A and S8B Fig), reflecting the predominant influence of genetic drift and mutation on heteroplasmy [49]. There were 31 out of 48 predicted pathogenic mutations that increased in DAF in mother- autistic proband pairs, in contrast to only 13 out of 36 predicted pathogenic mutations that increased in DAF in mother-sibling pairs (Fisher’s exact test, *P* = 0.015, S8E and S8F Fig). Taken together, these results indicated that the transmission pattern of heteroplasmies of high pathogenic potential differed between mother-autistic proband pairs and mother-sibling pairs, resulting in a greater abundance of these mutations on mtDNA in autistic children.

## Discussion

By employing a set of stringent criteria in calling heteroplasmies, we found a prevalence that 21.1% of individuals carry at least one medium-to-high fraction heteroplasmy with MAF of at least 5%. In order to draw meaningful comparisons with previous studies, we re-calculated the prevalence of heteroplasmy by applying the same MAF detection threshold at 5% in studies using blood specimens. In Rebolledo-Jaramillo *et al*.’s study, the prevalence of heteroplasmy is 30.8% among 39 children and 41.0% among their mothers [36]. Li *et al*. also identified a 41.0% prevalence of heteroplasmy in the blood specimens from 139 individuals with an average age of 58 [34]. Both studies employed mtDNA-targeted deep sequencing to sequence mtDNA to an extremely high coverage, yielding sufficient power for detecting heteroplasmies with MAF down to 1%. Using a similar design to our study, Ding *et al*. attained a 33.2% prevalence of heteroplasmy among 2,120 individuals with an average age of 47 years by analyzing the whole-genome sequencing data of SardiNIA Project participants [38]. A 180X depth of average coverage was obtained on mtDNA. Ding *et al*. also noticed a considerable elevation of heteroplasmy incidence among elderly Sardinians [38]. Hence, the prevalence of heteroplasmy among children and young adults could be lower than 33.2%, which was close to our estimate of heteroplasmy prevalence at 21.1%. A 20–30% prevalence of medium-to-high fraction heteroplasmies in blood was also verified by Sanger-based sequencing, showing that both the prevalence and frequency spectrum of mtDNA heteroplasmies were largely comparable to results generated by massively parallel sequencing [50].

The average depth of mtDNA coverage was significantly lower in mothers (132X [interquartile range, IQR:96X-150X], t-test, *P*<0.0003) than that in siblings (142X [IQR:103X-162X]) and probands (148X [IQR:105X-166X]). This effect could be related to age-dependent decrease of mtDNA copy number in blood [38], and could theoretically have influenced some results in the study. However, the difference of mtDNA coverage between probands and siblings in this dataset was minor and was not statistically significant (t-test, *P* = 0.074). To ensure such differences in mtDNA sequencing coverage did not confound our results, we performed a proof-of-concept analysis by down-sampling the complete dataset to harmonize sequencing coverage in the mother-proband-sibling trio of each family. In brief, we down-sampled reads of individuals to the lowest depth sequenced in the mother-proband-sibling trio at each mtDNA site. This procedure ensured that individuals from the same family had equal sequencing coverage at every mtDNA site, thus eliminating the possible influence of distinct sequencing coverage on heteroplasmy calling between different groups. Results from this analysis showed that a similar number of heteroplasmies were detected from down-sampled reads compared to total reads in all groups (S1 Text), indicating that lowering read counts in some individuals did not decrease power to identify heteroplasmy differences among family members. The difference between the complete and the down-sampled datasets among mothers, siblings, and probands was also comparable to each other (Fisher’s exact test, *P*>0.34 for pair-wise comparisons, S1 Text). Moreover, the enrichment of nonsynonymous and predicated pathogenic mutations in probands remained consistent in independent down-sampling analyses, close to the enrichment level that we computed based on total reads (S9 Fig). Down-sampling reads in each family also did not qualitatively alter the transmission pattern of heteroplasmies between mothers and children (S1 Text). Overall, this test indicated that sequencing coverage differences between mothers and children did not significantly bias heteroplasmy calling and comparisons in the current study.

An extremely high transition-to-transversion ratio was among *de novo* mutations in children: only 4 out of the 210 *de novo* heteroplasmic mutations were transversion mutations (three detected in probands and one detected in siblings). Such a mutation pattern is indicative of mtDNA replication errors and thus early developmental effects, rather than mtDNA damage caused by elevated oxidative stress associated with ASD [51]. Cell and animal studies [52,53] as well as mitochondrial pathophysiology [31] studies have demonstrated that genetic defects in OXPHOS genes culminate in elevated oxidative stress, inflammation, and metabolic abnormalities. However, the impact of oxidative stress on mtDNA may prevail among low-fraction heteroplasmies and mtDNA deletions. Further studies using mtDNA-targeted deep sequencing will enable us to systematically evaluate mtDNA mutation patterns in autistic patients.

We found that autistic probands carried overrepresented heteroplasmic mutations at non-polymorphic mtDNA sites, resulting in a ~1.5-fold enrichment of nonsynonymous mutations (*P* = 0.0028) as well as a ~2.2-fold enrichment of predicted pathogenic mutations (*P* = 0.0016) compared to those in non-autistic siblings (Fig 2C). The enrichment of predicted pathogenic mutations in autistic probands was not affected by the pathogenicity scores used and was further confirmed by the analysis of disease-associated mutations (S4 Fig). These results point to significant elevation of pathogenicity on mtDNA for autistic patients and thus provide a molecular basis for mitochondria-related pathophysiological phenotypes, as well as the high prevalence of MD among autistic patients [14,19].

In addition, we observed an overrepresented transmission of mtDNA mutations of high pathogenic potential from the mother to the autistic proband (Fig 3 and S2 Table). Due to the complete maternal inheritance of mtDNA [46,54], purifying selection in the maternal line may retain pathogenic mutations that possess a high mitochondrial threshold in females but a low mitochondrial threshold in males [55]. A recent study showed a male-biased expression profile for mitochondria-related genes during early childhood and puberty in the human brain [56]. Accordingly, pathogenic mtDNA mutations may adversely impact brain function when transmitted from the mother to the male proband, possibly contributing to the observed sexual dimorphism whereby males are more susceptible to neurodevelopmental disorders [57].

Of interest, such a transmission pattern of ASD-associated variants is also evidenced by the maternal transmission disequilibrium of nuclear LGD SNVs and rare CNVs in male autistic probands, but is inconclusive in female probands [24]. We assume that there can be a similar sex-dependent effect of pathogenic mtDNA heteroplasmies on ASD. Of the 903 families, there were 815 and 88 families with male and female probands, respectively, for a ratio of male:female of 9.3:1. A significant difference in transmission pattern was identified for nonsynonymous and predicted pathogenic mtDNA heteroplasmies in male probands compared to that in their siblings (ratio>1.3, one-tailed paired t-test, *P*<0.02, S2 Table), but no difference was present in families with female probands (ratio = 1, S2 Table). In contrast, comparable transmission patterns of mtDNA heteroplasmies to autistic probands were found when we stratified families according to the sibling sex. Given that the sample size of female probands was rather small in the current dataset, additional data on more ASD families with female probands would be required to confirm this finding and potentially reveal other differences in mtDNA heteroplasmy between families with male probands and families with female probands.

The majority (>65%) of mtDNA mutations detected in the current study had derived allele fraction in the range of 5% to 20%, below the clinical threshold for most disease-causing heteroplasmies [42]. It is still elusive how low-to-medium fraction heteroplasmies affect mitochondrial function. Picard *et al*. found that cells harboring the mtDNA m.3243A>G heteroplasmic mutation undergo a biphasic phenotypic change in response to the rise of heteroplasmy level; a medium heteroplasmy level (10%-30%) is presumed to cause mild OXPHOS deficiency in Diabetes [58] and Autism [59], while a high heteroplasmy level (50%-90%) gives rise to diverse, severe forms of neurodegenerative diseases such as mitochondrial encephalomyopathies [60]. Hence, individuals carrying a high degree of mtDNA heteroplasmies are prone to manifest systematic symptoms of MD rather than behavioral signs of ASD. Indeed, it is estimated that 10%-20% of MD patients have some autistic endophenotypes, further suggesting a possible, shared etiology for these two diseases [19].

Some neurological and developmental pathologies of MD [61] are present in ASD patients, including seizure, eating disorders, impaired motor ability and developmental delay [45,62,63]. Among the 33 autistic children detected with predicted pathogenic and/or disease-associated private mtDNA mutations in the current study, whose medical records were also available at SFARI phenotype database, six were reported with febrile or non-febrile seizures and two were diagnosed with epilepsy (S3 Table). One autistic child was found to have anorexia nervosa and the other two were reported with movement abnormalities (S3 Table). Early signs of metabolic or developmental defects related to MD [64] in these 33 autistic children included neonatal hypotonia (n = 3) and hypoglycemia (n = 3) (S3 Table), both of which were identified as risk factors for ASD [65,66]. Medical records also revealed a significantly higher incidence of mental retardation among the 33 autistic children than that in the rest of the database (Fisher’s exact test, *P* = 0.048, S3 Table), in line with our findings on the negative association between pathogenic mtDNA mutations and IQ. The overall prevalence of aforementioned abnormalities was 1.2 times higher in these 33 autistic children than other autistic children included in this study as well as in the rest of the SFARI database (36.4% vs. 16.7%, Fisher’s exact test, *P*<0.008, S3 Table). Furthermore, a decrease in height was found among these autistic children compared to other autistic children in the SFARI database (Mann-Whitney test, *P* = 0.026, S3 Table). Shorter stature was previously proposed as one of the major symptoms of MD [61,67] and was discovered in patients with mtDNA(m.3243A>G) heteroplasmy-caused diabetes (i.e. maternally inherited diabetes and deafness, MIDD) compared to non-MIDD diabetic patients such as patients diagnosed with type 1 or type 2 diabetes [68], suggesting a common underlying pathophysiology of mtDNA origin. Taken together, these evidence indicate that pathogenic mtDNA mutations may be responsible for a wide range of developmental abnormalities in children which confer susceptibility to ASD. It also calls attention to ASD children with one or more developmental abnormalities or related co-morbid clinical conditions for further testing on mtDNA and mitochondrial defects.

In sum, our findings support the connection between mtDNA variation and ASD, and call on further mtDNA-targeted deep sequencing and functional studies in more ASD families, which are of great importance to understand mtDNA mutation patterns and their contribution to ASD-related phenotypes. Indeed, mitochondrial dysfunction has been implicated in many childhood disorders, especially neurodevelopmental disorders [69]. These diseases manifest metabolic and physiological phenotypes that converge upon mitochondrial dysfunction, and may have mtDNA defects as a common harbinger. Further understanding the genetic architecture of mitochondrial genome in these phenotypes will provide crucial insights into pathogenesis, diagnosis, and treatment of these diseases.

## Materials and Methods

### Study samples and sequencing data

We downloaded the whole-exome sequencing data for 1,905 simplex families from the National Database for Autism Research (NDAR) under the study DOI:10.15154/1149697 (last accessed Nov. 2015). The raw sequencing data were generated by three genome centers including CSHL (n = 933), YALE (n = 599) and UW (n = 373). Only reads generated by CSHL had sufficient coverage on mtDNA (S1 Fig). We obtained similar results using alignment files generated from the same exome sequencing data set by Krumm *et al*. [24]. A possible explanation for such discrepancy of mtDNA coverage across different genome centers might lie in the different exome capturing protocol and sequencing read length used by each center [23,40]. Hence, we analyzed the 933 families sequenced at CSHL. We retrieved pertinent sample phenotypes including sex, ethnicity, age, full-scale intelligence quotient scores, Social Responsiveness Scale scores, and medical records from the supplementary data of previous publications on the SFARI Simons Collection [23,70], the sample information sheet available at NDAR, and/or the SFARI phenotype database (version 15).

### Sequencing data processing

We first extracted sequencing read pairs mapped to mtDNA from the downloaded alignment files. In order to filter out reads resulting from nuclear mitochondrial sequences (NUMTs), extracted reads were realigned to the complete human reference genome consisting of both human nuclear DNA and mtDNA sequences by using BWA (version 0.7.2) [71]. The reference sequences of the GRCh38 human genome assembly were obtained from the 1000Genomes project ftp site [41]. Reads mapped to the nuclear DNA were removed. Next, we performed a series of data processing steps to ensure the quality of remaining reads for calling mtDNA homoplasmies and heteroplasmies, including (1) local realignment around indels; (2) base quality recalibration; (3) detection and exclusion of duplicated reads, reads shorter than 40 bp, reads with ≥5% mismatches, as well as reads that were not aligned in a pair on mtDNA. The remaining reads were then piled up against the revised Cambridge Reference Sequence (rCRS) by using “samtool mpileup” with a mapping quality filter at 20 [72]. The list of tools and specific commands employed in each step was detailed in S10 Fig. A comparison of methods for heteroplasmy calling with previous studies was given in S4 Table.

### Point homoplasmy and heteroplasmy calling

To reduce the false positive rate, we only considered reads with a recalibrated base quality Phred score ≥20 (tantamount to ≤0.01 sequencing error) for calling point homoplasmies and heteroplasmies. For homoplasmy, the alternative allele according to rCRS must be supported by at least 10 reads and not be in the heteroplasmic state. Criteria for calling heteroplasmies encompassed (1) >40X depth of coverage, (2) the alternative allele being observed on both strands, and (3) comparable allele fractions calculated from reads mapped to two strands (Fisher’s exact test, *P*≥0.01). We also required that an mtDNA site was valid for calling heteroplasmies if its median site coverage was >70X depth in the study population. Based on one-tailed power calculation for one sample proportion test, it yielded >90% power of detecting real heteroplasmies with minor allele fraction (MAF) ≥ 5% over 1% sequencing error (S11 Fig). As a result, we obtained a total of 12,180 candidate heteroplasmies from 13,704 valid mtDNA sites (83% of total mtDNA sites, S5 Table and S12 Fig).

### Maximum likelihood based approach for estimating heteroplasmy fraction

Next, we utilized a maximum likelihood based approach to estimate MAF for each heteroplasmy by taking into account the base quality information of covered reads [37,40]. In brief, the likelihood of observing n reads with the minor allele and m reads with the major allele at a particular site was computed as:

$$L\;(f\;|\;mapped\;reads)=\prod_{i=1}^{n}((1-f)\varepsilon_{i}+f(1-\varepsilon_{i}))\;\prod_{i=1}^{m}\left((1-f)(1-\varepsilon_{i}\right)+f\;\varepsilon_{i})$$,

where f refers to the MAF of interest and *ε_i_* is the corresponding sequencing error for mapped read i. We then took the maximum-likelihood estimate of f and calculated the log likelihood ratio between the heteroplasmic model and the homoplasmic model (LLR = log(L($\hat{f}$)/L($\hat{f_{0}}$))). LLR score >5 was used as a criterion to define heteroplasmies of high confidence, equivalent to a false positive rate <10^−5^ [40].

Of heteroplasmies detected with MAF ≥5% at valid sites, over 98% had a LLR score over 5 (S13 Fig), which was consistent with our power calculation. In addition to insufficient sequencing depth, heteroplasmies with a low LLR score can also result from sequencing error or complications from nuclear mitochondrial sequences (NUMTs). Both give rise to a much lower transition-to-transversion (Ti/Tv) ratio compared to real heteroplasmies on mtDNA, which predominantly arise from mutations caused by endogenous, mtDNA polymerase replication errors [51]. When filtered at MAF ≥5%, high-confidence heteroplasmies yielded a Ti/Tv ratio of 29.5, in line with the hypothesized mutation pattern of mtDNA. High-confidence heteroplasmies could be recovered with the LLR score filter >5 down to MAF = 2% (greater than the base quality error threshold 1% used), which had a Ti/Tv ratio comparable to that of heteroplasmies with MAF ≥5% (22.8 vs. 29.5; chi-square test *P* = 0.5; S6 Table). But the majority of low-fraction heteroplasmies (MAF<5%) were probably missing due to insufficient sequencing depth.

### Sample quality control

We first removed 19 samples with average mtDNA coverage ≤40X depth. To minimize DNA contamination problems, we further removed samples (n = 19) if they had (1) >5 heteroplasmies located at polymorphic mtDNA sites in this population and/or (2) >50% of heteroplasmies detectable as homoplasmies in another sample, which also accounted for >50% of homoplasmic sites in that particular sample distinct from those in the sample under investigation. Contamination detection was based on high-confidence heteroplasmies with MAF ≥2%. 903 (97.1%) families with sequencing data from the proband, the sibling and the mother remained and were used for further analysis. The exome sequencing data from the father were processed using the same method but were only utilized for exploring data quality and detecting sample contamination. The average depth of mtDNA coverage in the remaining samples was ~141X, comparable to the coverage reported in previous studies using whole-exome or whole-genome sequencing data [38,39,73]. Comparisons of site-specific sequencing coverage for homoplasmic and heteroplasmic sites also showed strong correlations between individuals within the same family (*R*^2^>0.6, S14 Fig).

After sample quality control, we obtained 677 high-confidence heteroplasmies with MAF ≥5% in 903 families (S1 Dataset). We checked the mtDNA site for each of the 677 heteroplasmies within the family where it was found. 649 of them (95.9%) were located at mtDNA sites (n = 447) where all members from a family had >40X sequencing depth and thus were used for comparing incidence and studying transmission pattern of heteroplasmies (S1 Dataset). We further defined the major allele in a family, the allele with the highest fraction in the mother-proband-sibling trio, as the reference allele for each site on the consensus sequence, and considered a heteroplasmy inherited if the reference allele and the derived allele in the child were detected in the mother with fraction ≥2%. Otherwise we considered it *de novo* in the child.

### Annotation of mtDNA variants

mtDNA variants were annotated by using the ANNOVAR pipeline [74]. Pathogenicity predictions for all possible mtDNA nucleotide substitutions according to the revised Cambridge Reference Sequence (rCRS) were retrieved from the CADD database (version 1.3). The CADD score is a composite matric for deleteriousness of human mutations obtained through agglomerating annotation information from 63 distinct sources, which outperforms most popular pathogenicity predictors such as PolyPhen-2 and SIFT [75]. We followed the recommended cutoff for the scaled CADD score at 15 to define pathogenic mutations and used a more stringent cutoff at 20 for sensitivity testing. Alternative pathogenicity scores were also used for verification, including PolyPhen-2 [76], Mutation Assessor [77] and MutPred [78]. PolyPhen-2 scores and Mutation Assessor scores were extracted from the MitImpact2 database (version 2.4) [79], and MutPred scores were retrieved from a previous study [43]. All of them generated pathogenicity scores comparable to CADD scores (S15 Fig). A list of disease-associated, nonsynonymous and RNA mutations was compiled according to the MITOMAP website [80] and the ClinVar database [81] (last accessed Jan. 2016). Mutations with ambiguous annotations such as “unclear”, “conflicting reports” or “further studies needed”, or listed as “synergistic” or “secondary” on the MITOMAP website were not included. Both position and mutant allele information were used for defining mutations. In addition, we constructed a list of mutations which were predicted pathogenic by at least two of the five pathogenicity predictors: (1) CADD Phred score >15; (2) PolyPhen-2 possible and probable damaging; (3) Mutation Assessor medium and high impact; (4) MutPred score >0.6; (5) associated with disease. We relied on this list of mutations to replicate results from analyses using only CADD scores. Related results were denoted as “combined prediction” in S4 Fig, S5 Fig and S9 Fig.

### mtDNA haplogroups

The mtDNA haplogroup information was assessed by using HaploGrep2 [82]. The resulting distribution of macro-haplogroups among the 903 families was representative of that among the U.S. population [83] (S7 Table). The concordance rate between the mtDNA-inferred ancestral lineage and self-reported maternal ethnicity or race was >96.8% in non-admixed populations including non-Hispanic white (n = 654) and Asian (n = 49) (S7 Table), in close agreement with the concordance rate recently reported for the NHANES study [83] and among 588 mtDNA-sequenced individuals [84].

### Statistical testing

Incidence of mtDNA mutations between probands and siblings was compared at a family level by using paired t-test. Differences in transmission pattern in mother-autistic proband pairs and mother-sibling pairs were assessed by using Fisher’s exact test. Other statistical methods, such as correlation test, ANOVA, linear regression and logistic regression, were also used as indicated in the text. Statistical testing in this study was carried out by using R (version 3.2.2) and python (version 2.7.5)/scipy (version 0.16.1).

## Supporting Information

S1 Dataset Heteroplasmies detected in mother-proband-sibling trios. (XLSX)

S2 Dataset Incidence of homoplasmies. (XLSX)

S1 Table Contribution of private mtDNA mutations to ASD in families stratified according to both IQ and SRS scores. (XLSX)

S2 Table Transmission pattern of mtDNA heteroplasmies in families stratified by proband sex and sibling sex. (XLSX)

S3 Table Neurological and developmental defects in autistic children carrying pathogenic and/or disease-associated private mtDNA mutations. (XLSX)

S4 Table Comparison of methods for calling heteroplasmies with other studies. (XLSX)

S5 Table Valid mtDNA sites for calling heteroplasmies. (XLSX)

S6 Table Transition-to-transversion (Ti/Tv) ratio among candidate heteroplasmies. (XLSX)

S7 Table Distribution of mtDNA macro-haplogroups among the 903 families. (XLSX)

S1 Text Possible confounding factors in heteroplasmy calling and heteroplasmy comparisons between probands and siblings. (DOCX)

S1 Fig Average mtDNA coverage. (A) Average depth of sequencing coverage on mtDNA and (B) percentage of mtDNA sites sequenced to > 20X depth per sample for reads generated by CSHL (3,732 samples), YALE (2,396 samples) and UW (1,492 samples).
(TIFF)

S2 Fig Incidence of mtDNA homoplasmies and heteroplasmies. (A) Incidence of mtDNA homoplasmies. MAF: minor allele frequency of homoplasmies. Site-specific incidence of homoplasmies was given in S2 Dataset. (B, C) Incidence of mtDNA heteroplasmies with minor allele fraction ≥5% and confidence score >5.
(TIFF)

S3 Fig Relative mutation rates of mtDNA variants. The box plot of relative mutation rates for heteroplasmies at non-polymorphic sites, heteroplasmies at polymorphic sites and homoplasmies at non-heteroplasmic sites. The relative mutation rate for each nucleotide substitution on mtDNA was previously estimated by counting the number of times each nucleotide mutated independently in the phylogenetic tree representing the global human mtDNA variation [44]. The relative mutation rates for all observed substitutions as well as observed nonsynonymous substitutions were shown. Outliers were not depicted for clarity. Each nucleotide substitution was only counted at most once. ***P<7x10^-23^; **P<6x10^-3^; *P*: p values for Mann-Whitney test.
(TIFF)

S4 Fig Comparison of pathogenic mutation burden between autistic probands and non-autistic siblings. Mutation burden was calculated using mutations with DAF ≥5% at mtDNA sites where all members in a family had >40X sequencing depth. Error bars represent the standard error of the mean. NS: nonsynonymous mutations; CADD: mutations with CADD Phred score >15 or >20; PolyPhen-2: possible and probable damaging mutations predicted by PolyPhen-2; Mutation Assessor: medium and high impact mutations predicted by Mutation Assessor; MutPred: mutations with MutPred score >0.6; disease-associated: nonsynonymous or RNA mutations associated with disease according to the MITOMAP website and the ClinVar database; combined prediction: mutations predicted pathogenic in at least two of the five aforementioned pathogenicity categories (detailed in Materials and Methods). *P*: p values for one-tailed paired t-test.
(TIFF)

S5 Fig Differential ratio of pathogenic mtDNA mutations between probands and siblings under varying DAF thresholds. The ratio of mtDNA mutations between probands and siblings is shown in (A) for nonsynonymous mutations, in (B,C) for mutations predicted pathogenic with CADD Phred score >15 or >20, and in (D) for mutations predicted pathogenic in at least two of the five pathogenicity categories (detailed in Materials and Methods). The black circles in each figure refer to the observed ratios of mtDNA mutations between autistic probands and non-autistic siblings under varying thresholds of minimum DAF (from 2% to 8%) for defining mutations. The pink area represents 90% confidence interval of the same ratio estimated based on 10,000 bootstrap samples of the 903 families.
(TIFF)

S6 Fig Association between private mtDNA mutations and IQ. Associations of IQ with proband-private mtDNA mutations were computed in 638 probands of the major ethnic group among the 903 families who had a reported family ancestry of “white” or “more-than-one-race” and an mtDNA-haplogroup inferred maternal ancestry of “Europe”. Boxplots of IQ were depicted according to proband-private (**A**) mutations at non-polymorphic sites, (**B**) mutations at polymorphic sites, (**C**) nonsynonymous mutations, (**D,E**) mutations predicted pathogenic based on CADD Phred score >15 or >20, and (**F**) disease-associated mutations. The dashed red lines indicate a IQ of 70. Coefficients (Beta, **β**) and significance levels (*P*) of the associations between proband-private mtDNA mutations and IQ were given in the Table below the boxplots based on results from linear regression Model 1 (adjusting for age and sex) and Model 2 (adjusting for age, sex, SRS scores and whether carrying *de novo* LGD SNPs or CNVs on nuclear DNA).
(TIFF)

S7 Fig Proportion of inherited heteroplasmies in children. The proportion of heteroplasmies could be detected in the mother with DAF ≥2%.
(TIFF)

S8 Fig Changes of derived allele fractions of mtDNA mutations in mother-sibling pairs and mother-proband pairs. Changes of derived allele fractions (ΔDAF = DAF_child_−DAF_mother_) of mtDNA mutations were depicted in A, C, E, and G for mother-sibling pairs (blue), and in B, D, F, and H for mother-proband pairs (red). Only mtDNA sites (n = 448) detected with high-confidence heteroplasmies (MAF≥5%) or *de novo* homoplasmies were used for calculation. The total number of mutations used for plotting was indicated in parentheses under each figure. CADD: mutations with CADD Phred score >15 or >20.
(TIFF)

S9 Fig Differential ratio of pathogenic mtDNA mutations computed after down sampling reads in each family. The ratio of mtDNA mutations between probands and siblings is shown in (A) for nonsynonymous mutations, in (B,C) for mutations predicted pathogenic with CADD Phred score >15 or >20, and in (D) for mutations predicted pathogenic in at least two of the five pathogenicity categories (detailed in Materials and Methods). The open circles and lines in each figure indicate the ratios of mtDNA mutations between autistic probands and non-autistic siblings computed using total reads under varying thresholds of minimum DAF (from 2% to 8%) for defining mutations. The filled circles, squares, and triangles refer to the same ratios of mtDNA mutations between autistic probands and non-autistic siblings computed from three independent down-sampling procedures to harmonize mtDNA sequencing coverage in the mother-proband-sibling trio of each family. The statistical significance that the ratio is greater than one (*P* for one-tailed paired t-test) is shown using different colors indicated in the legend on the right side.
(TIFF)

S10 Fig Data processing workflow. (TIFF)

S11 Fig Power calculation for heteroplasmy calling. Results were obtained from one-tailed power calculation for one sample proportion test of discriminating real heteroplasmies from sequencing errors under the assumption of base quality error ≤1% (~0.33% for each possible substitution). Each curve refers to the power for detecting heteroplasmies with increasing MAF using the sequencing depth indicated in the legend.
(TIFF)

S12 Fig Genomic distribution of mtDNA heteroplasmies. The 677 high-confidence, median-to-high fraction heteroplasmies (MAF≥5%) were depicted with their positions on the mitochondrial genome indicated on the X axis, and their minor allele fractions indicated on the Y axis on the right hand side. Heteroplasmies detected in mothers, siblings, and probands were depicted using squares, triangles and diamonds, respectively. Heteroplasmies in grey are transitions and heteroplasmies in red are transversions. The black curve represents the median sequencing coverage for each mtDNA site as indicated on the Y-axis on the left hand side. Both of Y axes are on logarithmic scales. Fluctuation of sequencing coverage is due to complexity of the mitochondrial genome and its sequence similarity with baits designed for whole exome sequencing [40]. The middle panel displays the exact positions (black) of the 13,704 mtDNA sites valid for calling heteroplasmies. The lower panel displays the D-loop region (grey), the 24 RNA genes (blue), and the 13 protein-coding genes (red) on the mitochondrial genome.
(TIFF)

S13 Fig Distribution of confidence scores among candidate heteroplasmies. The 12,180 candidate heteroplasmies were detected from 13,704 mtDNA sites valid for calling heteroplasmies. The cumulative distributions of LLR scores for heteroplasmies were shown in the above figures stratified by the minor allele fraction.
(TIFF)

S14 Fig Correlations of site coverage at homoplasmic and heteroplasmic sites between individuals within each family. The regression lines were depicted in red.
(TIFF)

S15 Fig Comparisons between CADD predictions and other pathogenicity predictions on mtDNA mutations. For all predictors, the higher the pathogenicity score of a nucleotide substitution, the greater the chance it is pathogenic. ***P<2x10^-100^.
(TIFF)
