# Supplementary material for: Genetic Evidence for Elevated Pathogenicity of Mitochondrial DNA Heteroplasmy in Autism Spectrum Disorder
Source: PLoS Genet. 2016 Oct 28;12(10):e1006391. doi: 10.1371/journal.pgen.1006391 (PMC5085253; doi:10.1371/journal.pgen.1006391)
Supplement: S1 Text — (DOCX) [file pgen.1006391.s010.docx]

**Genetic Evidence for Elevated Pathogenicity of Mitochondrial DNA Heteroplasmy in Autism Spectrum Disorder**

Yiqin Wang^1^, Martin Picard^2^, and Zhenglong Gu^1^

^1^ Division of Nutritional Sciences, Cornell University, Ithaca, New York, United States of America

^2^ Department of Psychiatry, Division of Behavioral Medicine, and Department of Neurology, Division of Columbia Translational Neuroscience Initiative, Columbia University Medical Center, New York, New York, United States of America

**Supporting information**

**Possible confounding factors in heteroplasmy calling and heteroplasmy comparisons between probands and siblings**

**mtDNA sequencing coverage**

In the 903 families included in the current study, the average depth of mitochondrial DNA (mtDNA) coverage was significantly lower in mothers (132X [interquartile range, IQR:96X-150X], t-test, *P*<0.0003) than that in siblings (142X [IQR:103X-162X]) and probands (148X [IQR:105X-166X]). Although the difference of mtDNA coverage between probands and siblings in this dataset was minor (4% in average mtDNA coverage) and was not statistically significant (t-test, *P*=0.074), it might impact sensitivity of heteroplasmy identification, especially identification of low-fraction heteroplasmies, leading to a spurious association of mtDNA heteroplasmies with Autism Spectrum Disorder (ASD). To ensure the observed differences in mtDNA sequencing coverage did not confound our results, we first compared the frequency spectrum of identified mtDNA heteroplasmies in mothers, siblings and probands. If the observed difference in mtDNA coverage did confound the heteroplasmy calling in our study, we would expect to see an inflation of low-to-median fraction heteroplasmies in probands compared to those in siblings and mothers. But the fraction spectrum of heteroplasmies did not differ significantly among these groups (median/mean values of heteroplasmy fraction: 0.14/0.21 in siblings, 0.13/0.20 in probands, 0.13/0.19 in mothers; Mann-Whitney test, *P* > 0.17 for pair-wise comparisons). Moreover, there was little increase in the overall number of heteroplasmies (Fig 2A) detected in probands compared to that in siblings, suggesting that the 4% difference in sequencing coverage was unlikely to skew heteroplasmy identification in probands versus that in siblings.

To adjust for sequencing coverage differences between siblings and probands as well as between mothers and children, we tested a down-sampling method to harmonize sequencing coverage in the mother-proband-sibling trio of each family. In brief, we down-sampled reads of individuals to the lowest depth sequenced in the mother-proband-sibling trio at each mtDNA site. This procedure ensured that individuals from the same family had equal sequencing coverage at every mtDNA site, thus eliminating the possible influence of distinct sequencing coverage on heteroplasmy calling between different groups. We then performed variant calling and comparisons on the down-sampled dataset. We observed similar number of heteroplasmies detected from down-sampled reads compared to heteroplasmies detected from total reads in all groups (Table ST1), indicating that lowering read counts in some individuals did not decrease power to identify heteroplasmy differences between family members. The difference between the complete and the down-sampled datasets among mothers, siblings, and probands was also comparable to each other (Fisher’s exact test, *P*>0.34 for pair-wise comparisons, Table ST1). Overall, this test indicated that small sequencing coverage differences between siblings and probands as well as between mothers and children did not significantly bias heteroplasmy calling.

**Table ST1. mtDNA heteroplasmies called using down-sampled reads and total reads**

|  | Heteroplasmies called using | | Difference |
| --- | --- | --- | --- |
| Groups | Down-sampled reads | Total reads |  |
| Mother | 241 | 246 | -5 (-1.9%) |
| Sibling | 227 | 230 | -3 (-1.2%) |
| Proband | 235 | 242 | -7 (-2.8%) |

The data on down-sampled reads are averages over three independent down-sampling procedures.

Next, we used the down-sampled dataset to perform the same analyses presented in the main text to compare incidence of heteroplasmies between probands and siblings, as well as their transmission pattern from mothers to children. Again, the down-sampling procedure did not qualitatively alter our observations from analyses based on total reads; the enrichment of non-synonymous and predicated pathogenic mutations in probands remained consistent in independent down-sampling analyses, close to the enrichment level that we computed based on total reads (S9 Fig). Down-sampling reads in each family also did not alter the transmission pattern of heteroplasmies between mothers and children compared to results calculated using total reads (Table ST2).

Taken together, these analyses indicated that the difference in sequencing coverage in probands, siblings and mothers was not a source of bias, and confirmed the validity of our results and conclusions.

**Table ST2. Transmission pattern of mtDNA heteroplasmies called using down-sampled reads and total reads**

|  |  | #hetero-plasmies | Nonsynonymous (%) | CADD > 15 (%) | CADD > 20 (%) |
| --- | --- | --- | --- | --- | --- |
| Mother-Sibling | Untransmitted | 112 (110) | 30.1% (31.8%) | 15.5% (16.4%) | 12.8% (13.6%) |
|  | Transmitted | 130 (136) | 23.1% (22.8%) | 5.4% (5.1%) | 3.9% (3.7%) |
|  | *De novo* | 99 (95) | 22.6% (23.2%) | 10.5% (11.6%) | 8.8% (9.5%) |
| Mother-Proband | Untransmitted | 123 (120) | 21.7% (22.5%) | 8.7% (9.2%) | 6.8% (7.5%) |
|  | Transmitted | 119 (126) | 31.2% (31.0%) | 11.5% (11.1%) | 9.3% (8.7%) |
|  | *De novo* | 117 (116) | 32.3% (32.8%) | 18.3% (19.8%) | 13.1% (14.7%) |

Data are the number or the proportion of mtDNA heteroplasmies in each category computed based on down-sampled reads and total reads. Data for down-sampled reads are averages over three independent down-sampling procedures. Data for total reads are indicated in parentheses.

**Ages of probands and siblings**

Several previous studies reported that mtDNA heteroplasmy pathogenicity and incidence accumulate with age, especially in elderly population [1,2], but no evidence for the correlation between age and mtDNA heteroplasmies was reported in children. In the 903 families included in the analysis, we found a slight but statistically significant difference in ages of autistic probands and their siblings: the average ages of probands and siblings were 9.2 years (sd=3.5) and 9.9 years (sd=4.4), respectively (paired t-test, *P*=1x10^-7^). There was no significant association between age and detected heteroplasmies in neither probands nor siblings (linear regression, *P*>0.22). Furthermore, adjustment for age using conditional logistic regression did not result in qualitative changes in the odds ratio (OR) and the significance level of the associations between ASD and mtDNA heteroplasmies (Table ST3). The age-adjusted ORs calculated using conditional logistic regression (Table ST3) were close to the ORs given in Table 1. As such, age should not be a confounding factor that impacted the associations between ASD and mtDNA heteroplasmies.

**Table ST3. Associations of mtDNA mutations with ASD with and without age as a covariate.**

|  | Model 1 (without age adjustment) | | Model 2 (adjusting for age) | |
| --- | --- | --- | --- | --- |
| Group | OR [95% CI] | *P* | OR [95% CI] | *P* |
| All | 1.06 [0.86-1.32] | 0.58 | 1.02 [0.82-1.27] | 0.87 |
| Nonsynonymous | 1.85 [1.19-2.89] | 0.0066 | 1.81 [1.16-2.85] | 0.0097 |
| CADD > 15 | 2.58 [1.34-4.97] | 0.0047 | 2.47 [1.27-4.80] | 0.0074 |
| CADD > 20 | 2.55 [1.20-5.43] | 0.015 | 2.42 [1.13-5.18] | 0.023 |

Odds ratio (OR), 95% confidence interval (CI) of OR, and significance level (P) of associations between ASD and mtDNA heteroplasmy incidence were computed using condition logistic regression (clogit) in the R “survival” package with and without age as a covariate. Model 1: logit(P(ASD)) ~ heteroplasmy + strata(family). Model 2: logit(P(ASD)) ~ heteroplasmy + age + strata(family); CADD: predicted pathogenic mtDNA mutations with CADD Phred score >15 or >20.

**References**

1. Ding J, Sidore C, Butler TJ, Wing MK, Qian Y, Meirelles O, et al. Assessing mitochondrial DNA variation and copy number in lymphocytes of ~2,000 Sardinians using tailored sequencing analysis tools. PLoS Genet. 2015;11: 1–18. doi:10.1371/journal.pgen.1005306

2. Williams SL, Mash DC, Züchner S, Moraes CT. Somatic mtDNA mutation spectra in the aging human putamen. PLoS Genet. 2013;9: e1003990. doi:10.1371/journal.pgen.1003990
