# Supplementary figures and images for: Genetic Evidence for Elevated Pathogenicity of Mitochondrial DNA Heteroplasmy in Autism Spectrum Disorder
Source: PLoS Genet. 2016 Oct 28;12(10):e1006391. doi: 10.1371/journal.pgen.1006391 (PMC5085253; doi:10.1371/journal.pgen.1006391)

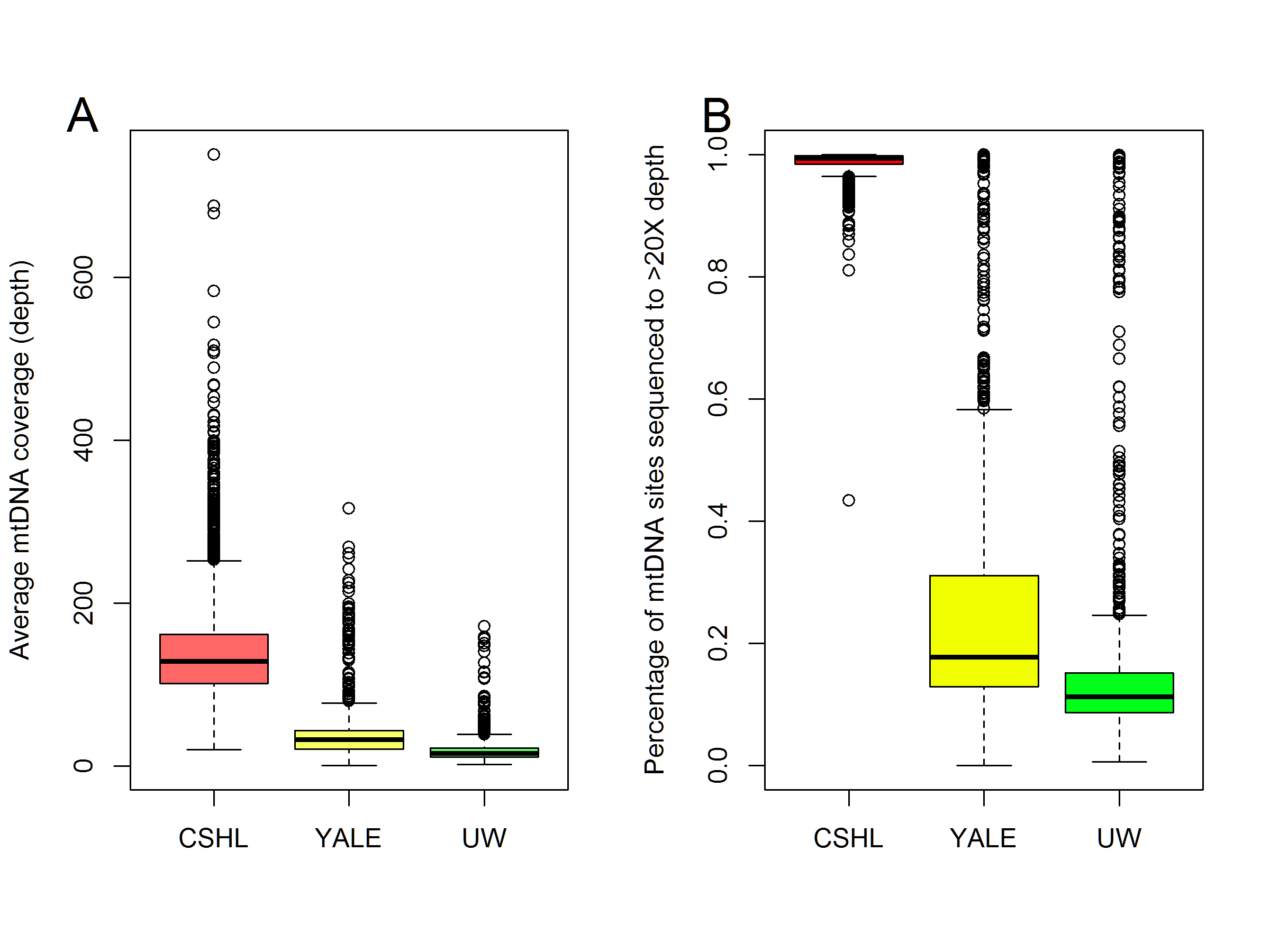

Supplement: S1 Fig — (A) Average depth of sequencing coverage on mtDNA and (B) percentage of mtDNA sites sequenced to > 20X depth per sample for reads generated by CSHL (3,732 samples), YALE (2,396 samples) and UW (1,492 samples). (TIFF) [file pgen.1006391.s011.tiff]

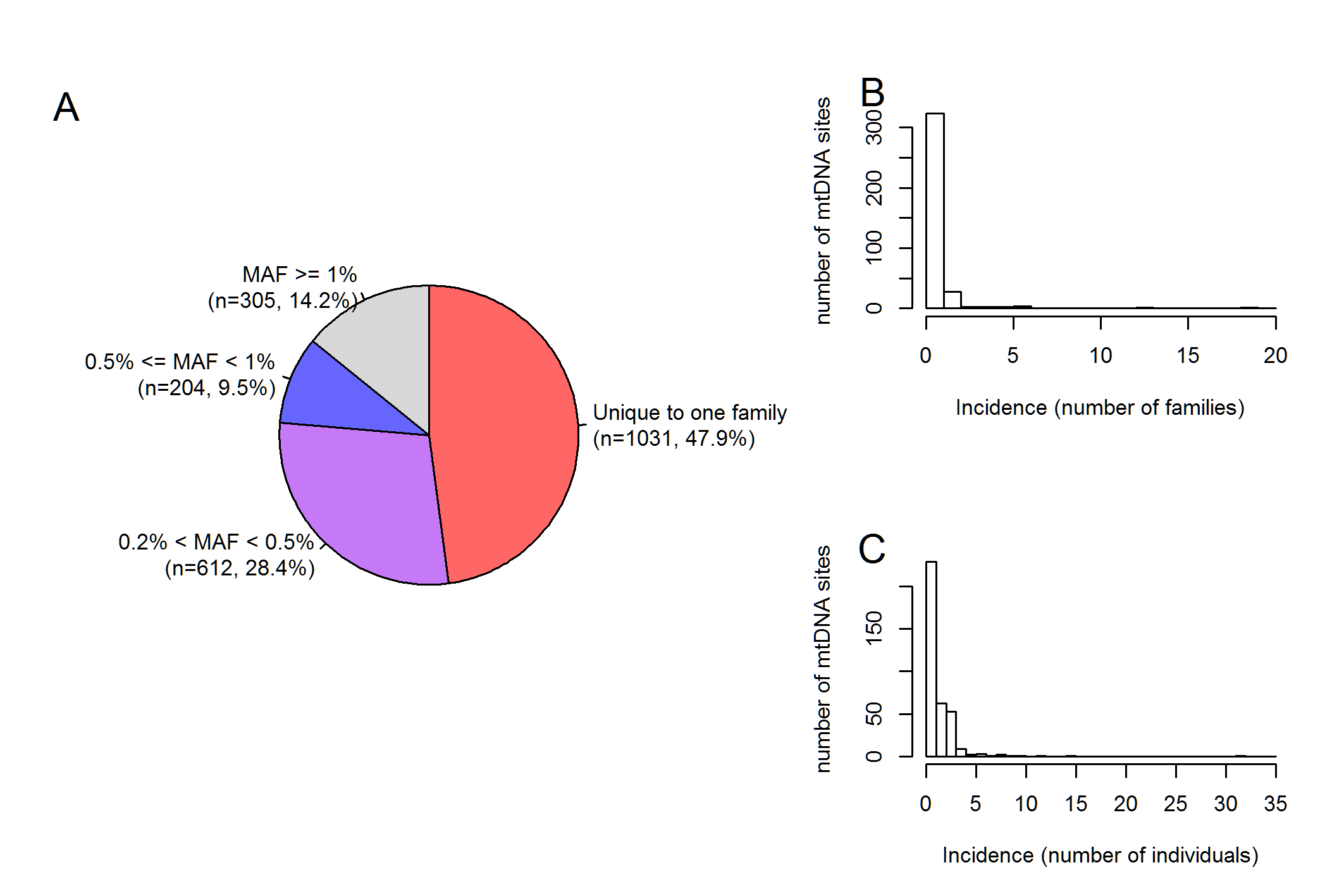

Supplement: S2 Fig — (A) Incidence of mtDNA homoplasmies. MAF: minor allele frequency of homoplasmies. Site-specific incidence of homoplasmies was given in S2 Dataset. (B, C) Incidence of mtDNA heteroplasmies with minor allele fraction ≥5% and confidence score >5. (TIFF) [file pgen.1006391.s012.tiff]

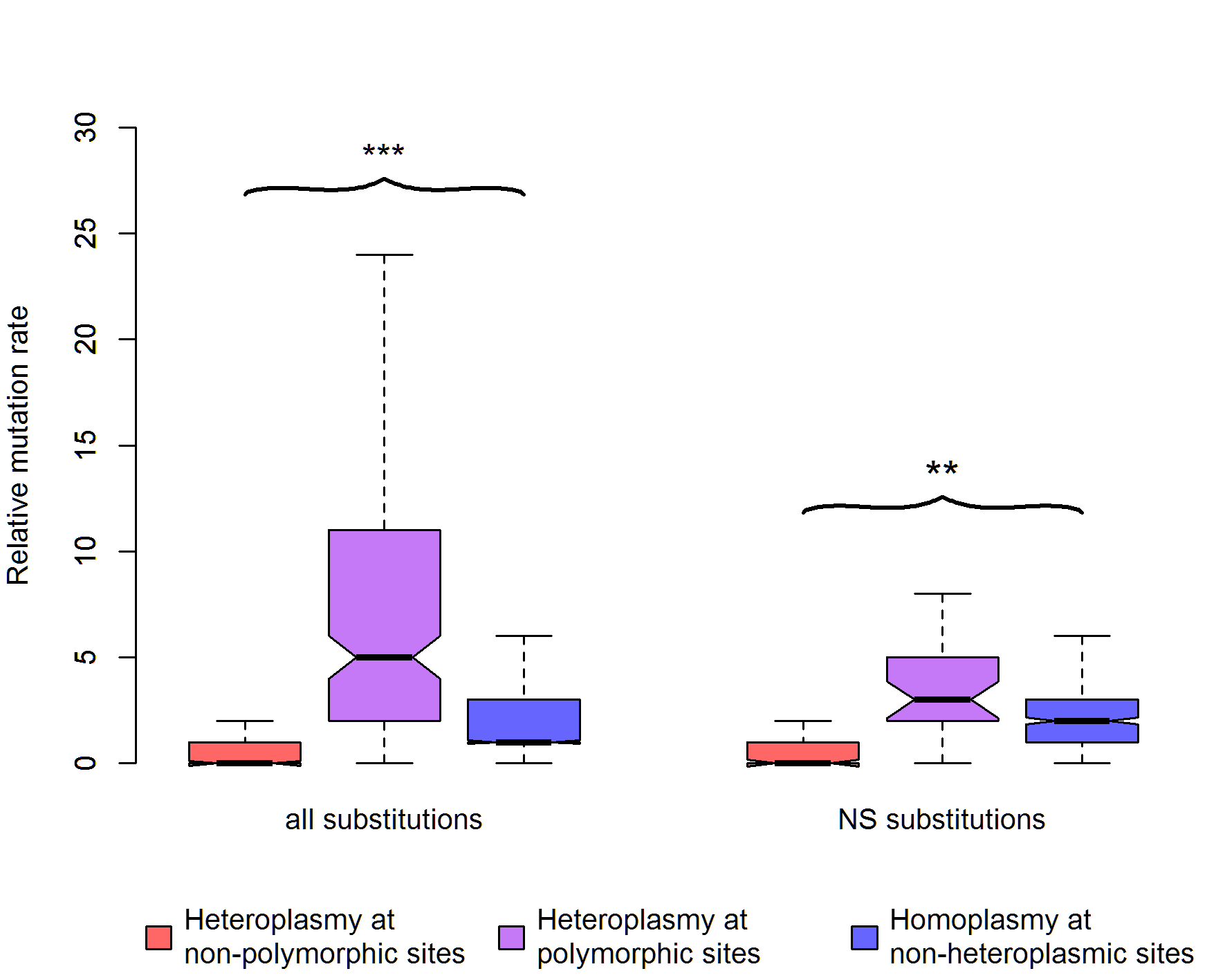

Supplement: S3 Fig — The box plot of relative mutation rates for heteroplasmies at non-polymorphic sites, heteroplasmies at polymorphic sites and homoplasmies at non-heteroplasmic sites. The relative mutation rate for each nucleotide substitution on mtDNA was previously estimated by counting the number of times each nucleotide mutated independently in the phylogenetic tree representing the global human mtDNA variation [44]. The relative mutation rates for all observed substitutions as well as observed nonsynonymous substitutions were shown. Outliers were not depicted for clarity. Each nucleotide substitution was only counted at most once. ***P<7x10^-23^; **P<6x10^-3^; P: p values for Mann-Whitney test. (TIFF) [file pgen.1006391.s013.tiff]

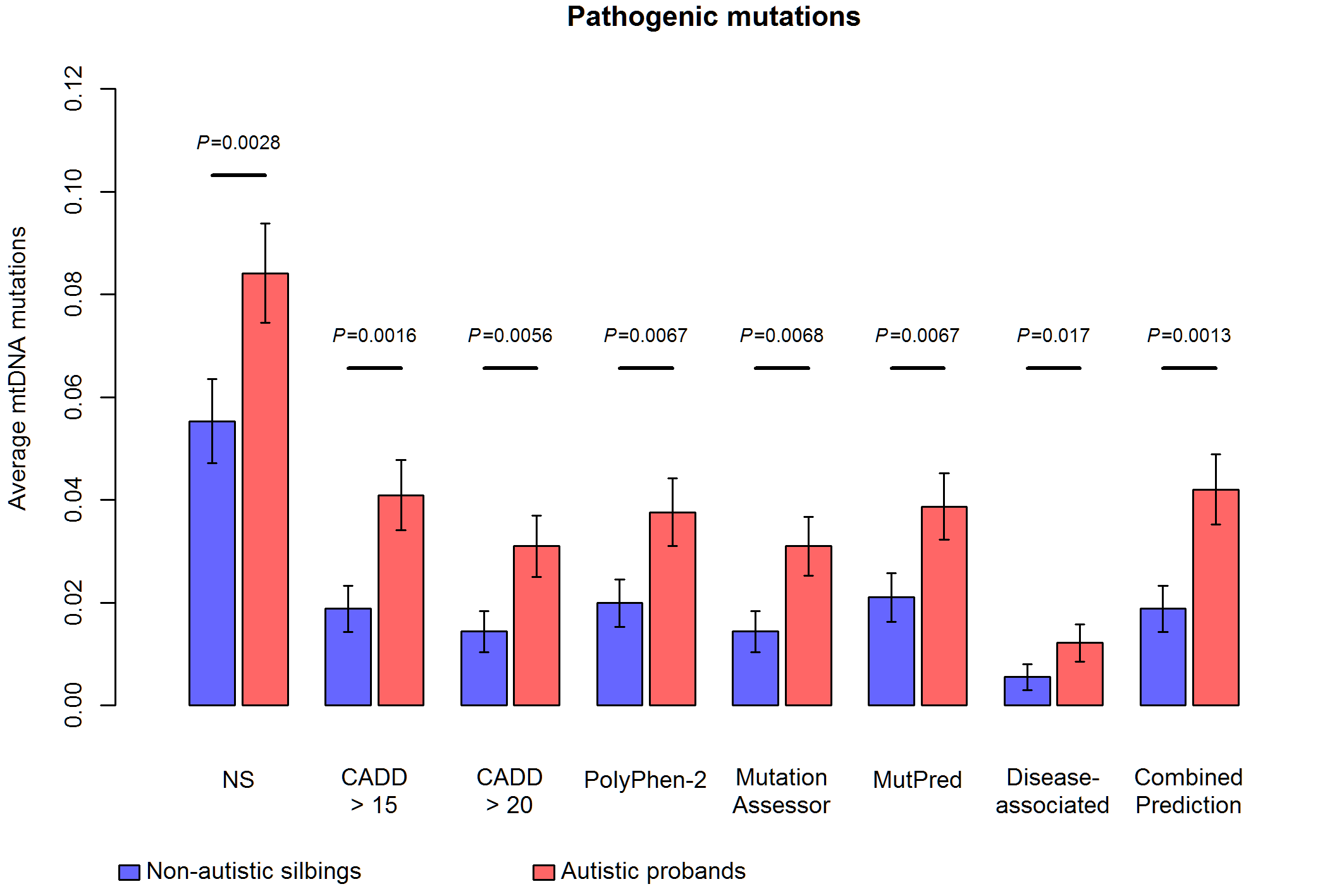

Supplement: S4 Fig — Mutation burden was calculated using mutations with DAF ≥5% at mtDNA sites where all members in a family had >40X sequencing depth. Error bars represent the standard error of the mean. NS: nonsynonymous mutations; CADD: mutations with CADD Phred score >15 or >20; PolyPhen-2: possible and probable damaging mutations predicted by PolyPhen-2; Mutation Assessor: medium and high impact mutations predicted by Mutation Assessor; MutPred: mutations with MutPred score >0.6; disease-associated: nonsynonymous or RNA mutations associated with disease according to the MITOMAP website and the ClinVar database; combined prediction: mutations predicted pathogenic in at least two of the five aforementioned pathogenicity categories (detailed in Materials and Methods). P: p values for one-tailed paired t-test. (TIFF) [file pgen.1006391.s014.tiff]

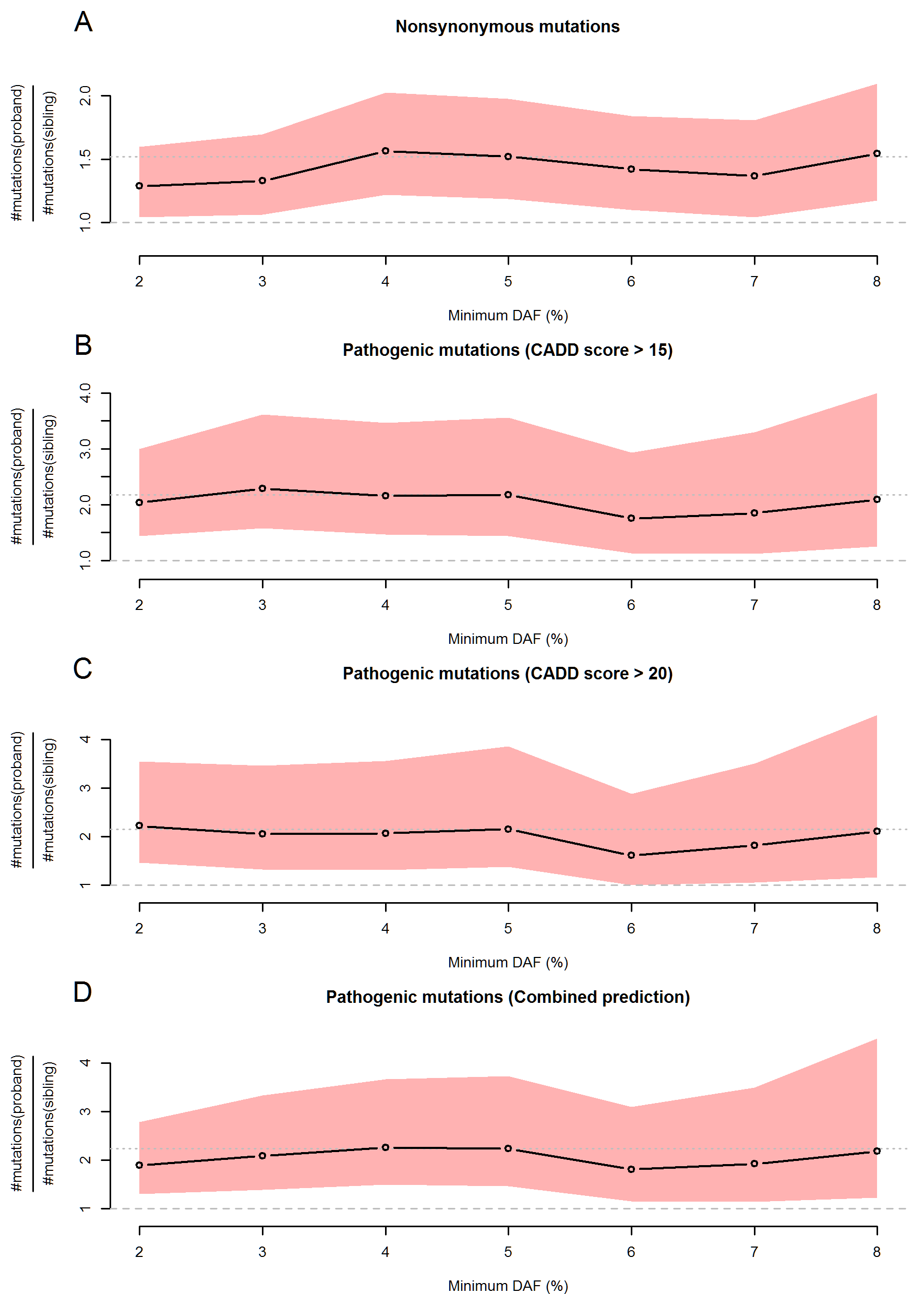

Supplement: S5 Fig — The ratio of mtDNA mutations between probands and siblings is shown in (A) for nonsynonymous mutations, in (B,C) for mutations predicted pathogenic with CADD Phred score >15 or >20, and in (D) for mutations predicted pathogenic in at least two of the five pathogenicity categories (detailed in Materials and Methods). The black circles in each figure refer to the observed ratios of mtDNA mutations between autistic probands and non-autistic siblings under varying thresholds of minimum DAF (from 2% to 8%) for defining mutations. The pink area represents 90% confidence interval of the same ratio estimated based on 10,000 bootstrap samples of the 903 families. (TIFF) [file pgen.1006391.s015.tiff]

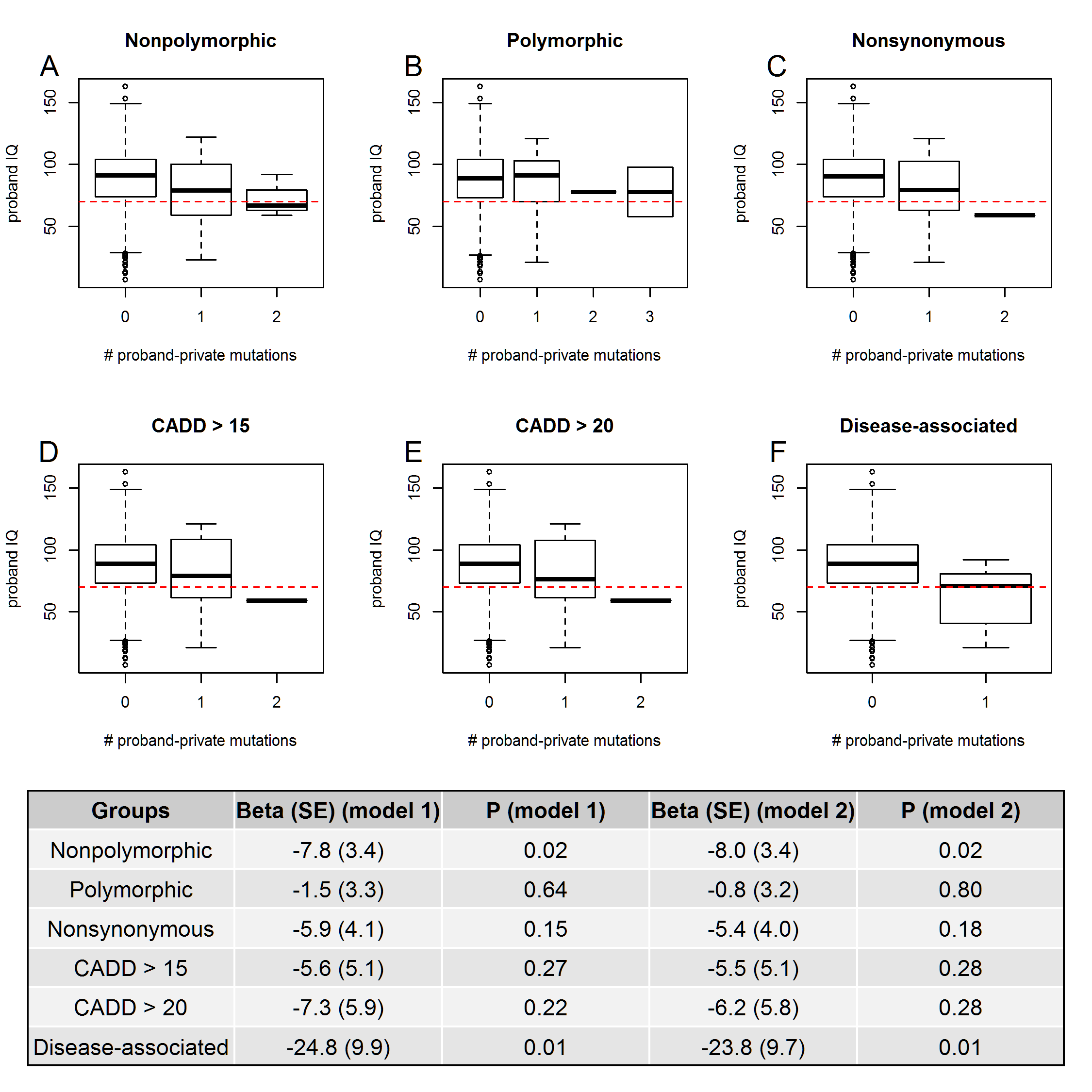

Supplement: S6 Fig — Associations of IQ with proband-private mtDNA mutations were computed in 638 probands of the major ethnic group among the 903 families who had a reported family ancestry of “white” or “more-than-one-race” and an mtDNA-haplogroup inferred maternal ancestry of “Europe”. Boxplots of IQ were depicted according to proband-private (A) mutations at non-polymorphic sites, (B) mutations at polymorphic sites, (C) nonsynonymous mutations, (D,E) mutations predicted pathogenic based on CADD Phred score >15 or >20, and (F) disease-associated mutations. The dashed red lines indicate a IQ of 70. Coefficients (Beta, β) and significance levels (P) of the associations between proband-private mtDNA mutations and IQ were given in the Table below the boxplots based on results from linear regression Model 1 (adjusting for age and sex) and Model 2 (adjusting for age, sex, SRS scores and whether carrying de novo LGD SNPs or CNVs on nuclear DNA). (TIFF) [file pgen.1006391.s016.tiff]

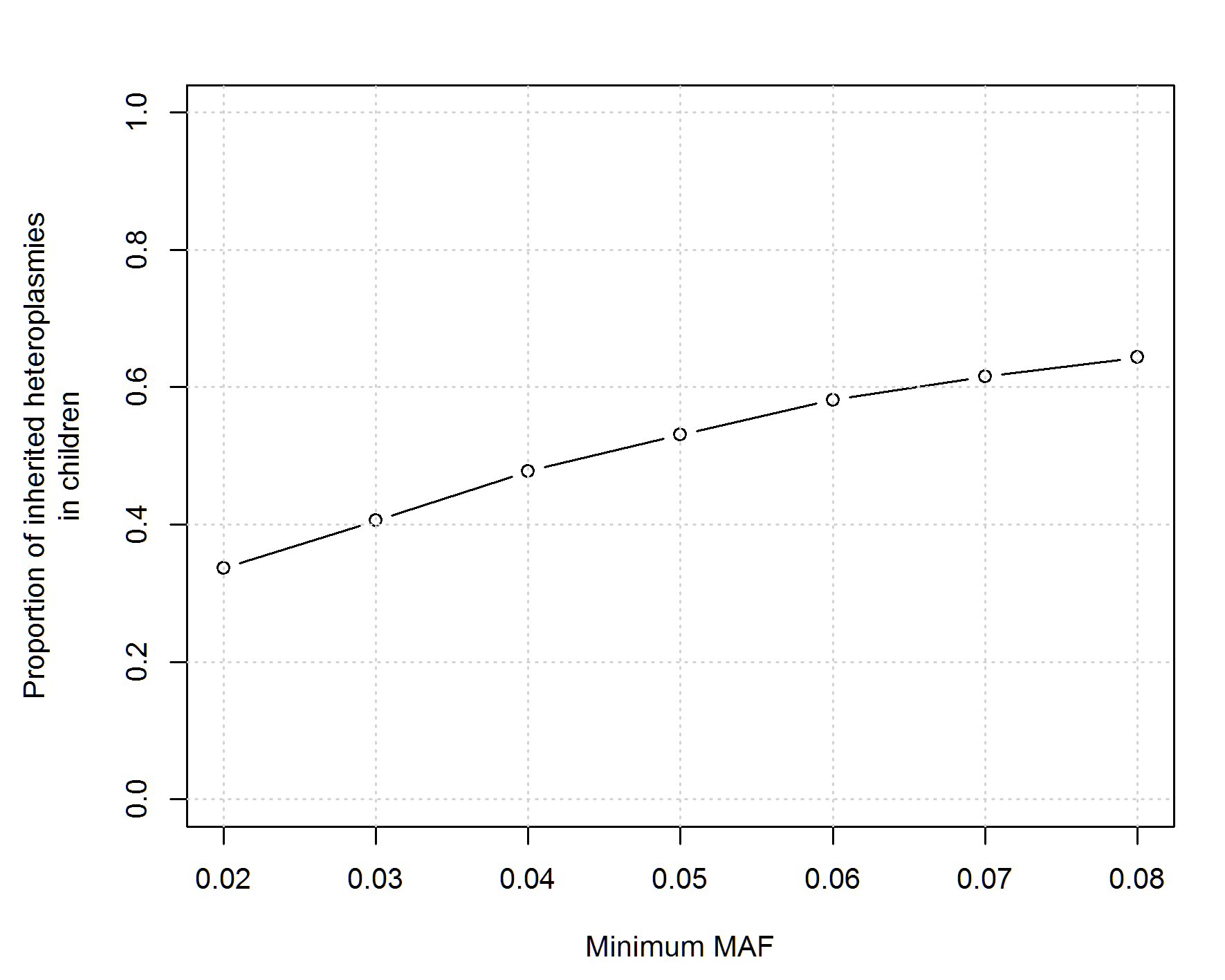

Supplement: S7 Fig — The proportion of heteroplasmies could be detected in the mother with DAF ≥2%. (TIFF) [file pgen.1006391.s017.tiff]

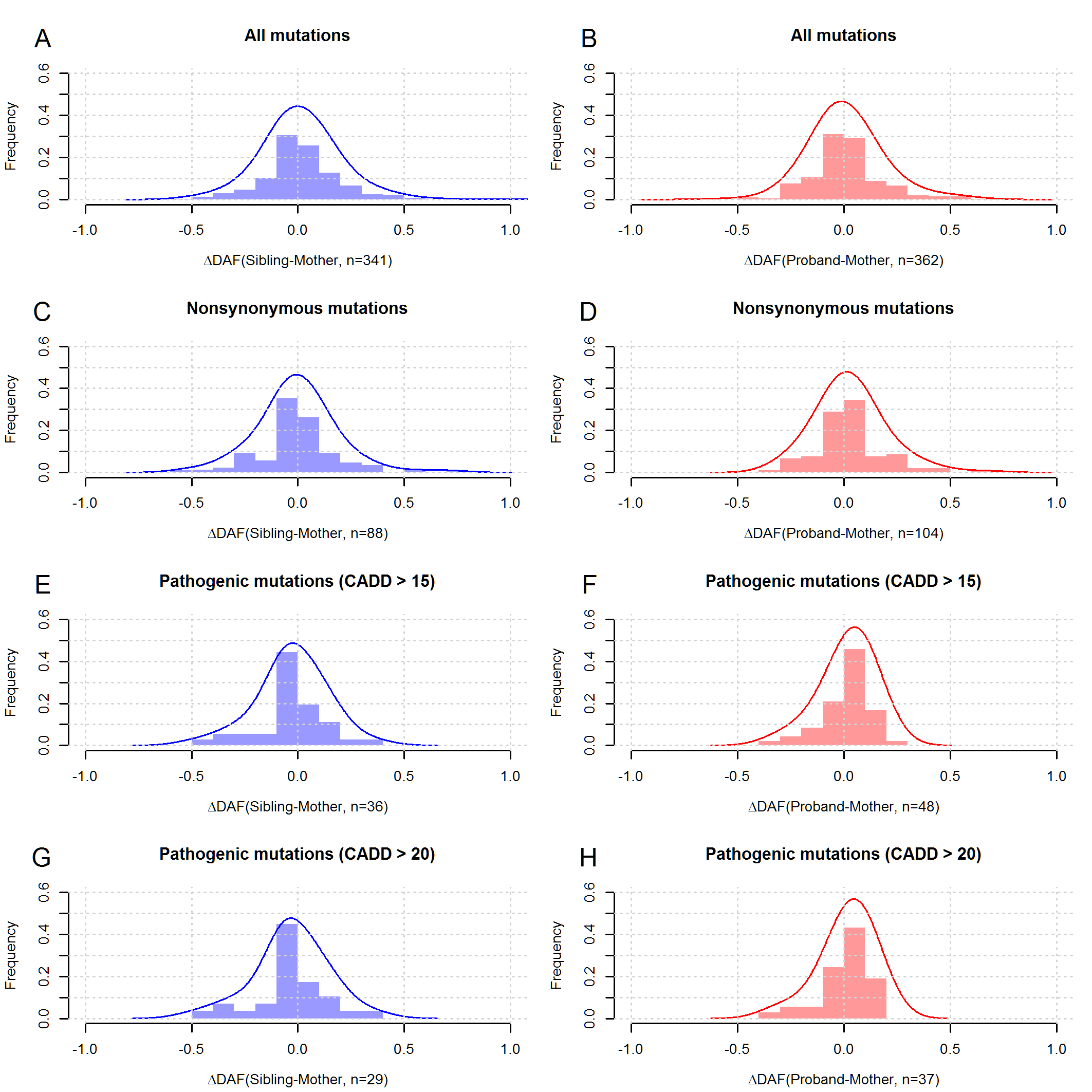

Supplement: S8 Fig — Changes of derived allele fractions (ΔDAF = DAFchild−DAFmother) of mtDNA mutations were depicted in A, C, E, and G for mother-sibling pairs (blue), and in B, D, F, and H for mother-proband pairs (red). Only mtDNA sites (n = 448) detected with high-confidence heteroplasmies (MAF≥5%) or de novo homoplasmies were used for calculation. The total number of mutations used for plotting was indicated in parentheses under each figure. CADD: mutations with CADD Phred score >15 or >20. (TIFF) [file pgen.1006391.s018.tiff]

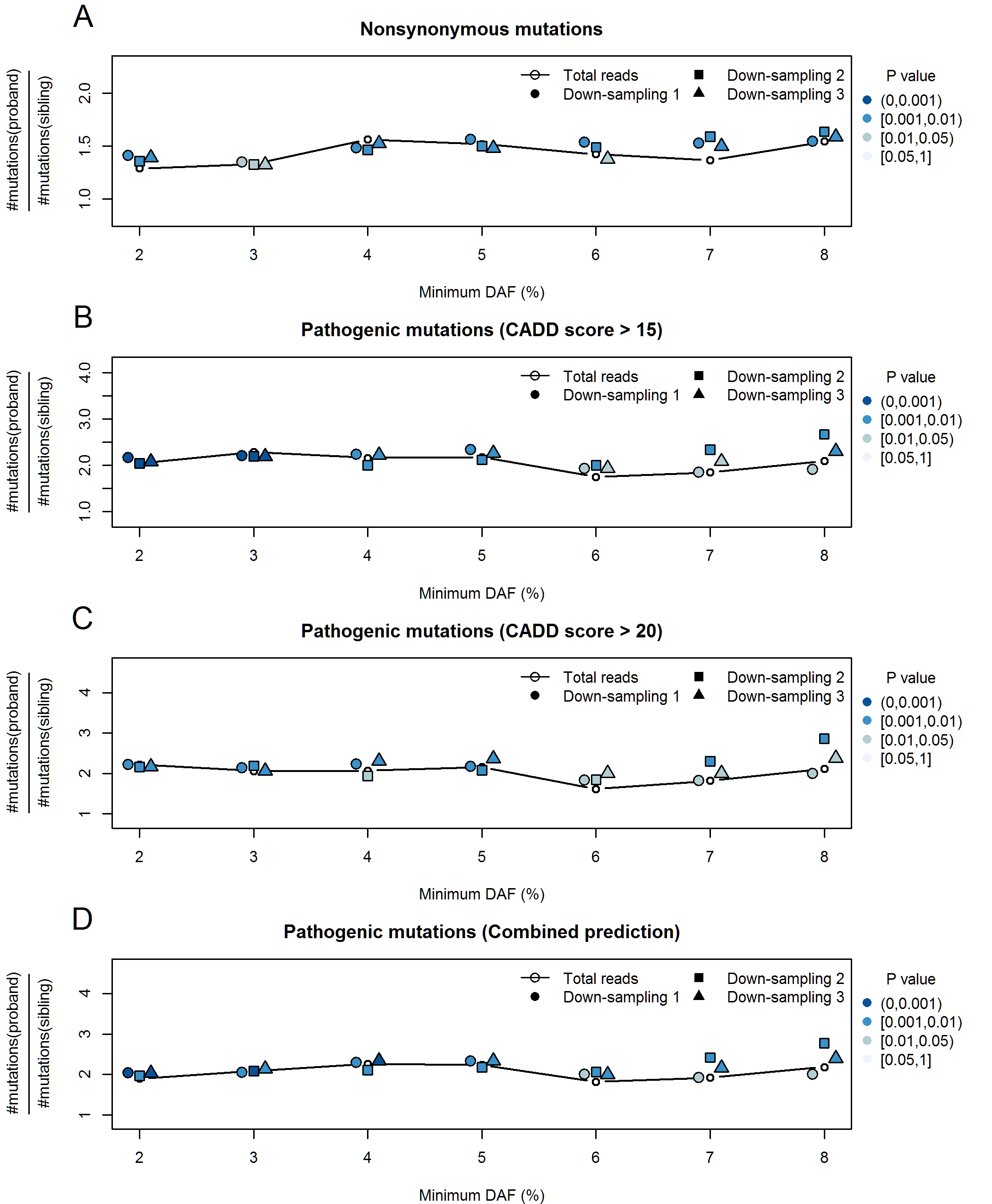

Supplement: S9 Fig — The ratio of mtDNA mutations between probands and siblings is shown in (A) for nonsynonymous mutations, in (B,C) for mutations predicted pathogenic with CADD Phred score >15 or >20, and in (D) for mutations predicted pathogenic in at least two of the five pathogenicity categories (detailed in Materials and Methods). The open circles and lines in each figure indicate the ratios of mtDNA mutations between autistic probands and non-autistic siblings computed using total reads under varying thresholds of minimum DAF (from 2% to 8%) for defining mutations. The filled circles, squares, and triangles refer to the same ratios of mtDNA mutations between autistic probands and non-autistic siblings computed from three independent down-sampling procedures to harmonize mtDNA sequencing coverage in the mother-proband-sibling trio of each family. The statistical significance that the ratio is greater than one (P for one-tailed paired t-test) is shown using different colors indicated in the legend on the right side. (TIFF) [file pgen.1006391.s019.tiff]

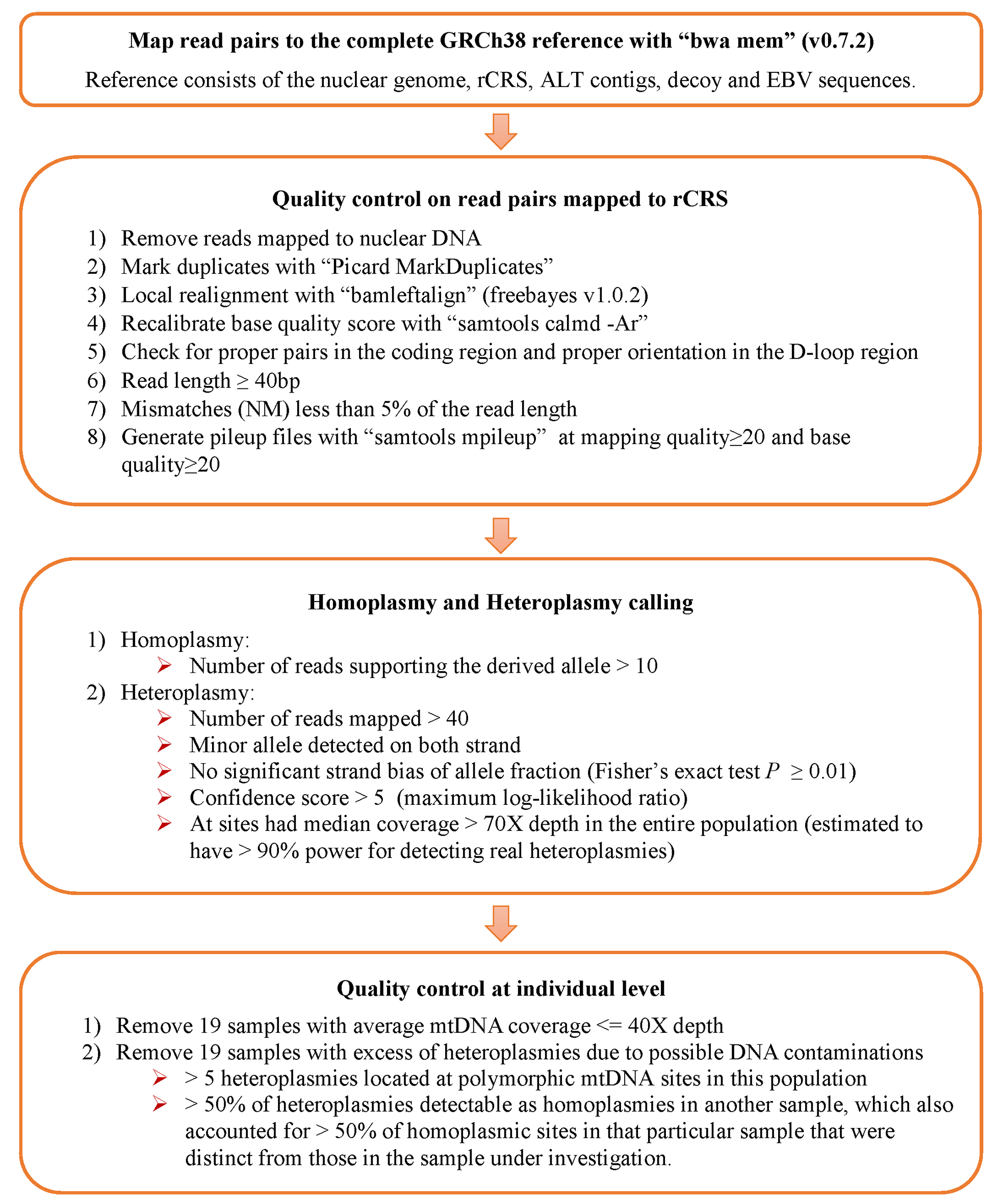

Supplement: S10 Fig — (TIFF) [file pgen.1006391.s020.tiff]

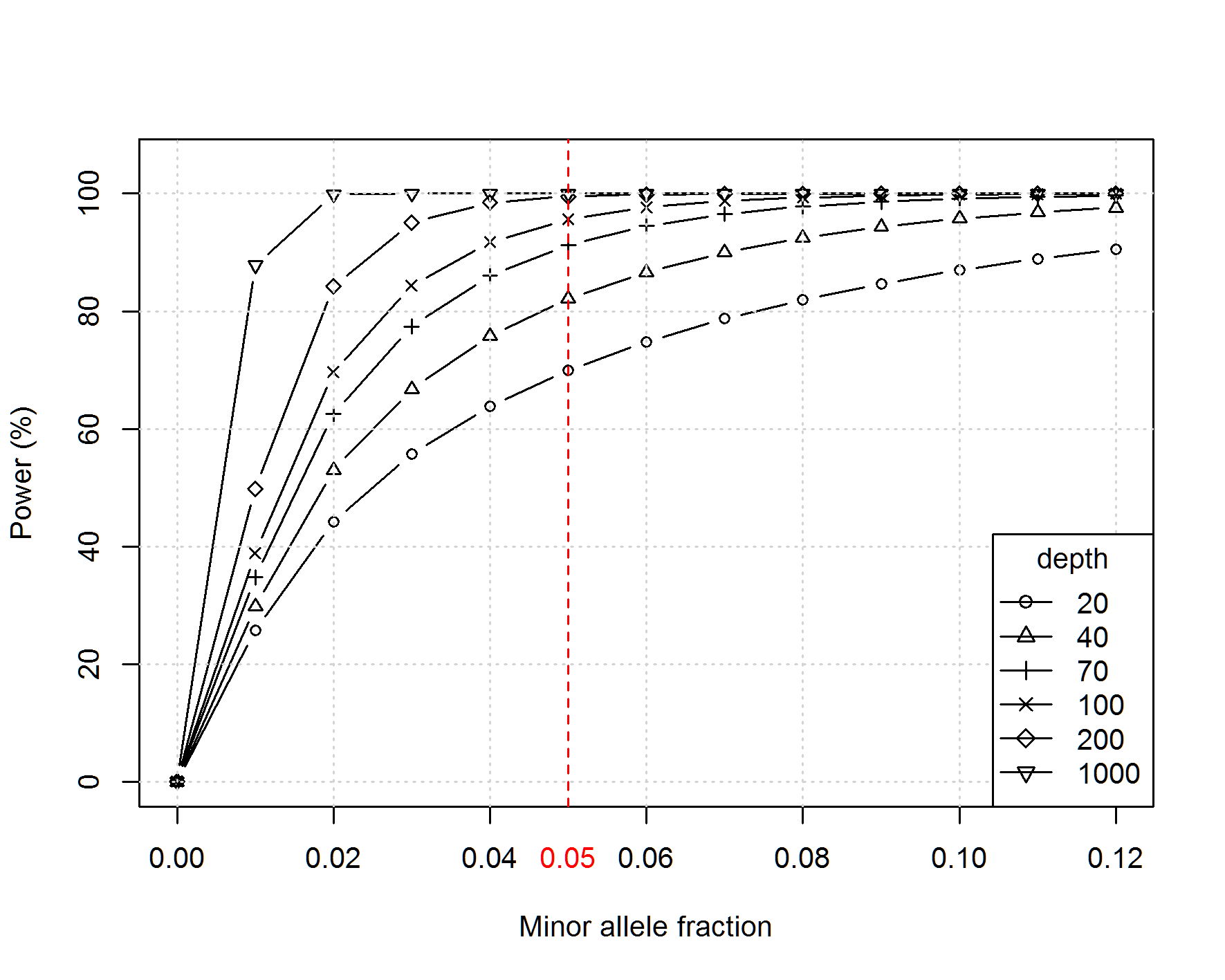

Supplement: S11 Fig — Results were obtained from one-tailed power calculation for one sample proportion test of discriminating real heteroplasmies from sequencing errors under the assumption of base quality error ≤1% (~0.33% for each possible substitution). Each curve refers to the power for detecting heteroplasmies with increasing MAF using the sequencing depth indicated in the legend. (TIFF) [file pgen.1006391.s021.tiff]

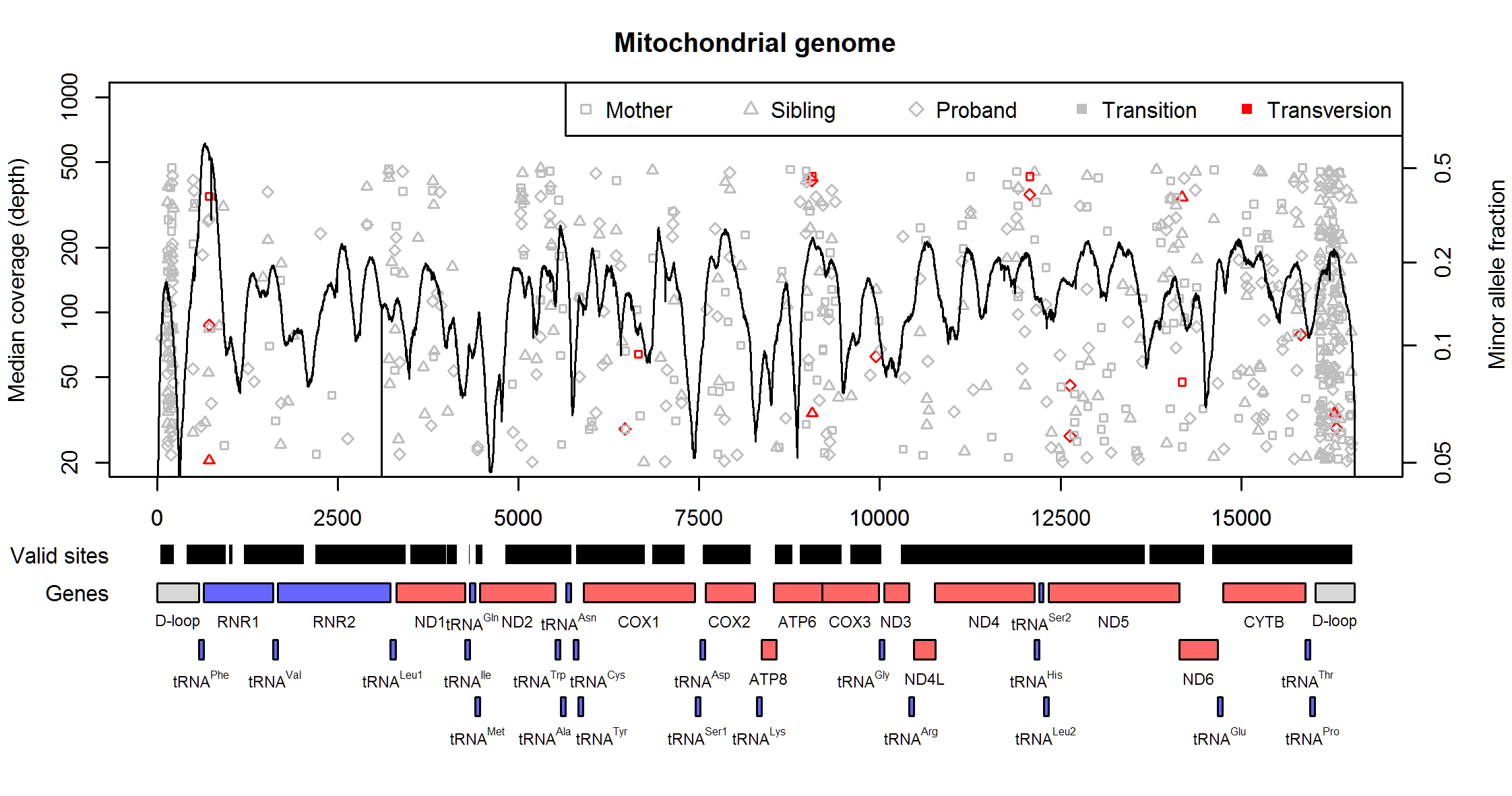

Supplement: S12 Fig — The 677 high-confidence, median-to-high fraction heteroplasmies (MAF≥5%) were depicted with their positions on the mitochondrial genome indicated on the X axis, and their minor allele fractions indicated on the Y axis on the right hand side. Heteroplasmies detected in mothers, siblings, and probands were depicted using squares, triangles and diamonds, respectively. Heteroplasmies in grey are transitions and heteroplasmies in red are transversions. The black curve represents the median sequencing coverage for each mtDNA site as indicated on the Y-axis on the left hand side. Both of Y axes are on logarithmic scales. Fluctuation of sequencing coverage is due to complexity of the mitochondrial genome and its sequence similarity with baits designed for whole exome sequencing [40]. The middle panel displays the exact positions (black) of the 13,704 mtDNA sites valid for calling heteroplasmies. The lower panel displays the D-loop region (grey), the 24 RNA genes (blue), and the 13 protein-coding genes (red) on the mitochondrial genome. (TIFF) [file pgen.1006391.s022.tiff]

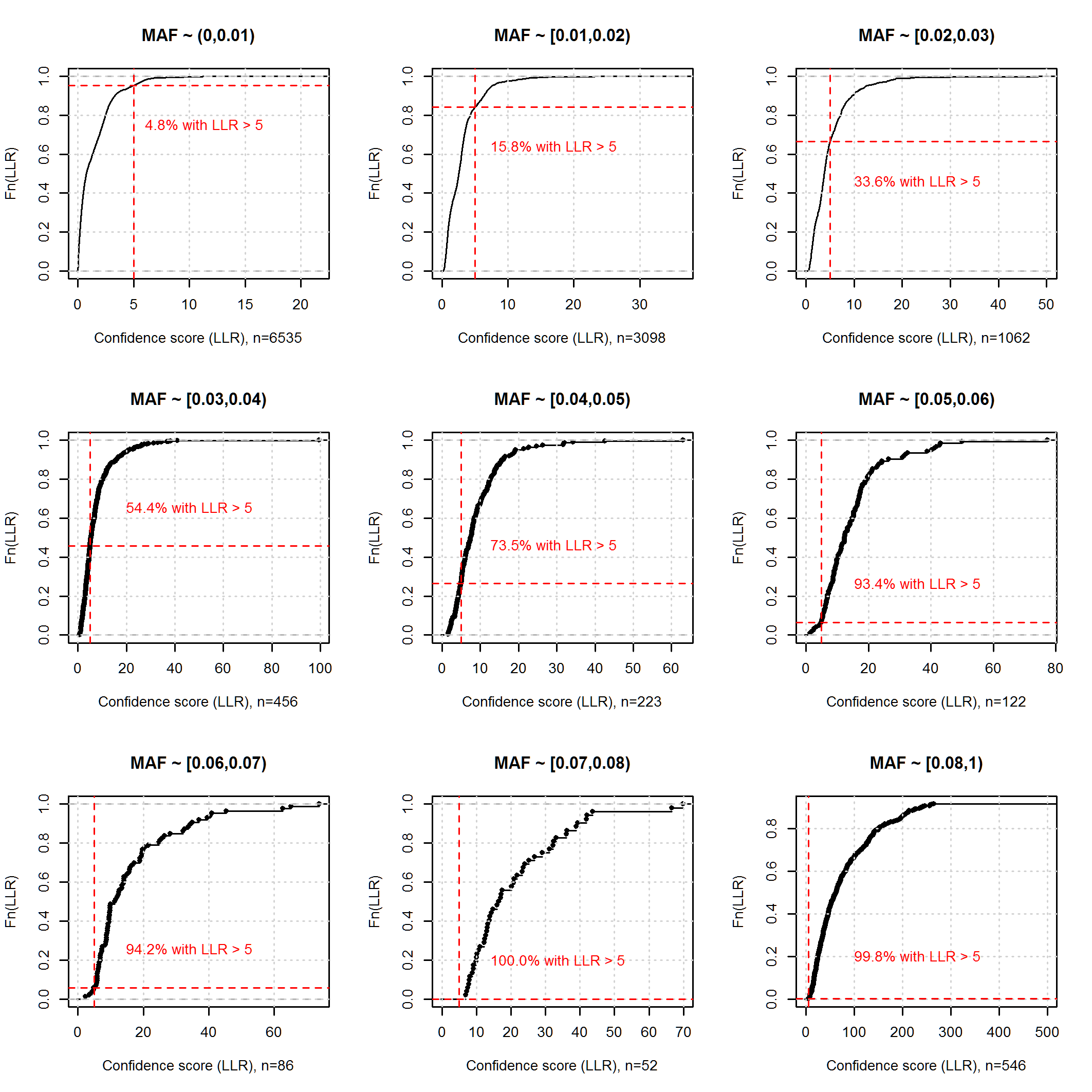

Supplement: S13 Fig — The 12,180 candidate heteroplasmies were detected from 13,704 mtDNA sites valid for calling heteroplasmies. The cumulative distributions of LLR scores for heteroplasmies were shown in the above figures stratified by the minor allele fraction. (TIFF) [file pgen.1006391.s023.tiff]

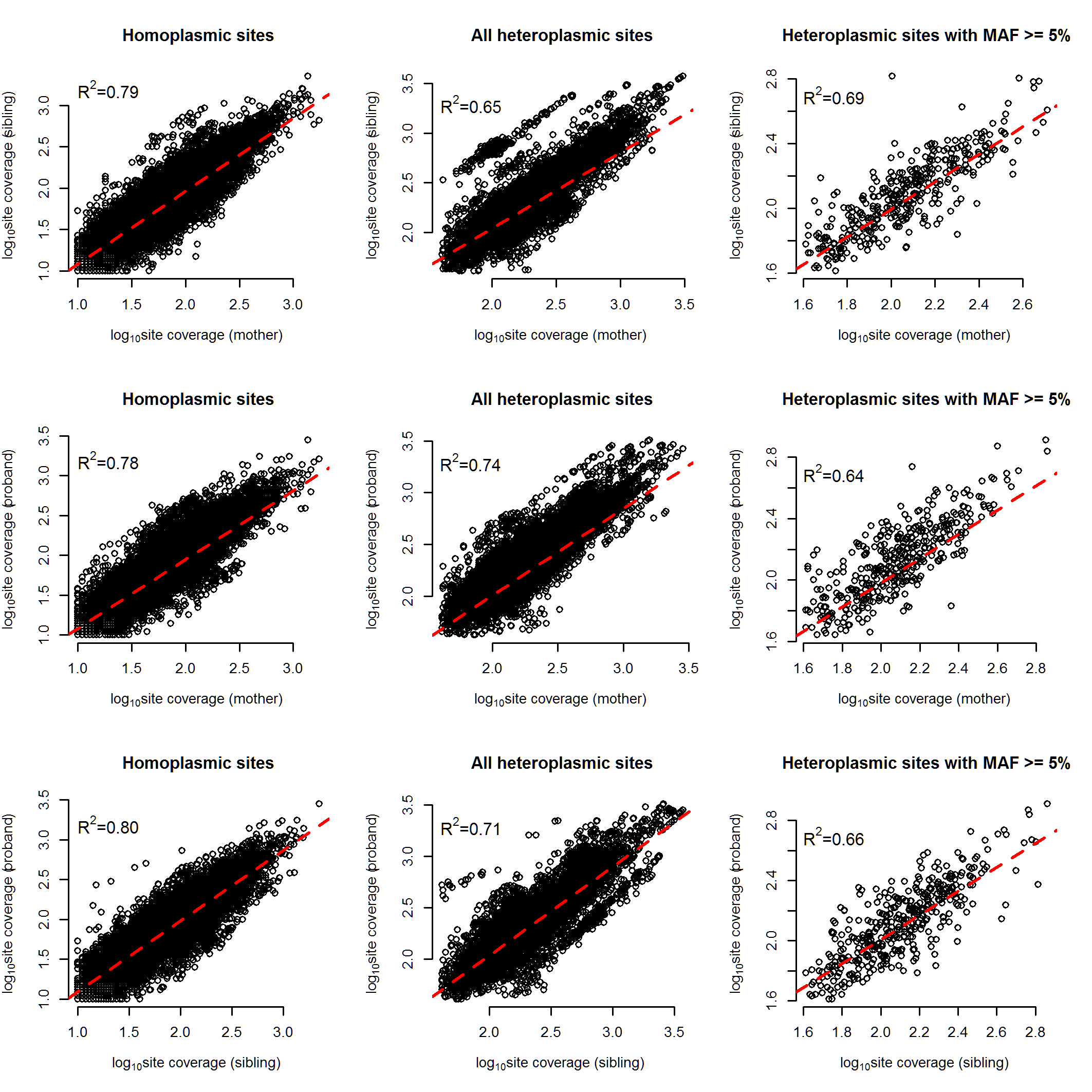

Supplement: S14 Fig — The regression lines were depicted in red. (TIFF) [file pgen.1006391.s024.tiff]

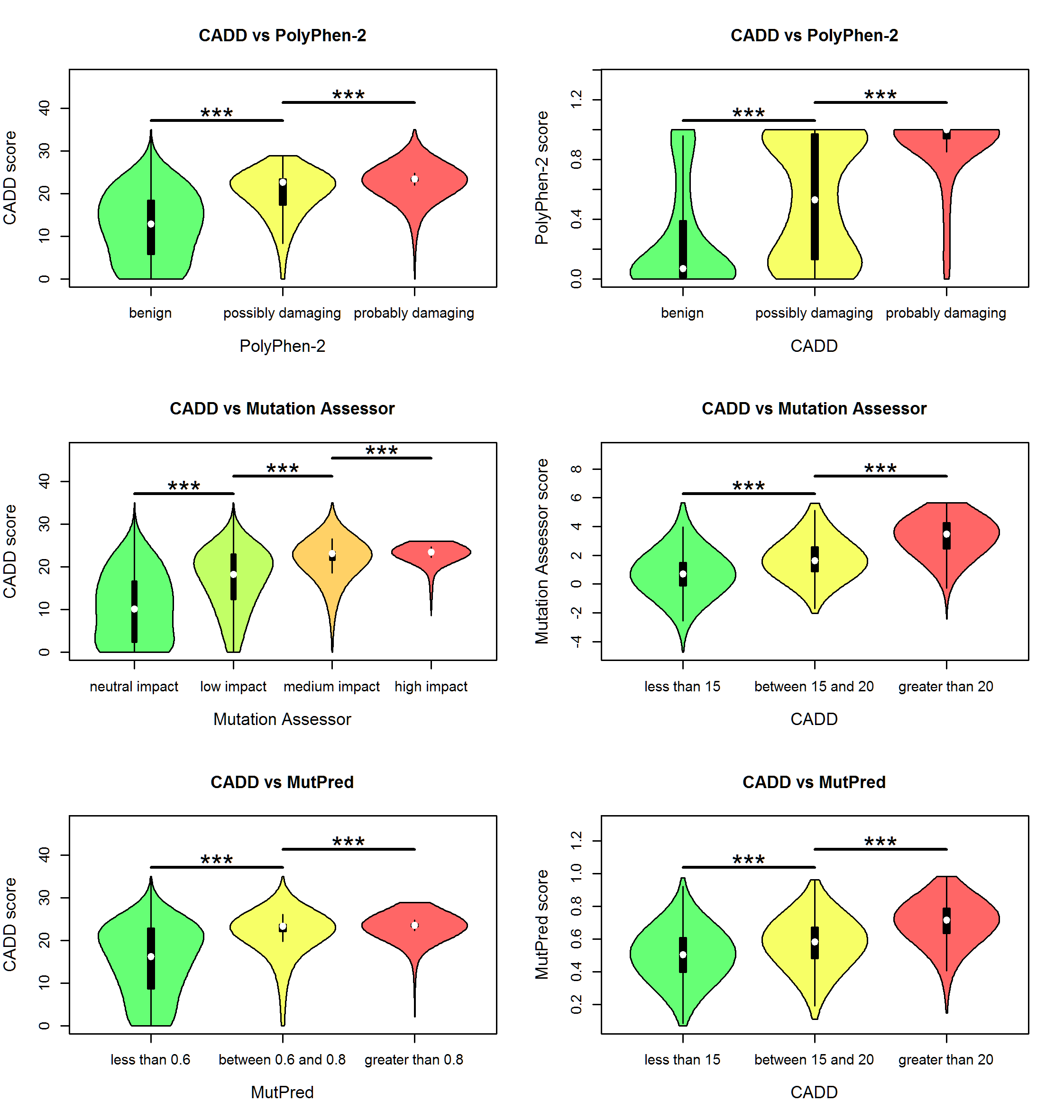

Supplement: S15 Fig — For all predictors, the higher the pathogenicity score of a nucleotide substitution, the greater the chance it is pathogenic. ***P<2x10-100. (TIFF) [file pgen.1006391.s025.tiff]
